# The structure of APOBEC1 and insights into its RNA and DNA substrate selectivity

**DOI:** 10.1093/narcan/zcaa027

**Published:** 2020-10-09

**Authors:** Aaron D Wolfe, Shuxing Li, Cody Goedderz, Xiaojiang S Chen

**Affiliations:** Genetics, Molecular and Cellular Biology, Keck School of Medicine, University of Southern California, Los Angeles, CA 90089, USA; Molecular and Computational Biology, Department of Biological Sciences, University of Southern California, Los Angeles, CA 90089, USA; Molecular and Computational Biology, Department of Biological Sciences, University of Southern California, Los Angeles, CA 90089, USA; Center of Excellence in NanoBiophysics, University of Southern California, Los Angeles, CA 90089, USA; Molecular and Computational Biology, Department of Biological Sciences, University of Southern California, Los Angeles, CA 90089, USA; Genetics, Molecular and Cellular Biology, Keck School of Medicine, University of Southern California, Los Angeles, CA 90089, USA; Molecular and Computational Biology, Department of Biological Sciences, University of Southern California, Los Angeles, CA 90089, USA; Center of Excellence in NanoBiophysics, University of Southern California, Los Angeles, CA 90089, USA; Norris Comprehensive Cancer Center, University of Southern California, Los Angeles, CA 90089, USA

## Abstract

APOBEC1 (APO1), a member of AID/APOBEC nucleic acid cytosine deaminase family, can edit apolipoprotein B mRNA to regulate cholesterol metabolism. This APO1 RNA editing activity requires a cellular cofactor to achieve tight regulation. However, no cofactors are required for deamination on DNA by APO1 and other AID/APOBEC members, and aberrant deamination on genomic DNA by AID/APOBEC deaminases has been linked to cancer. Here, we present the crystal structure of APO1, which reveals a typical APOBEC deaminase core structure, plus a unique well-folded C-terminal domain that is highly hydrophobic. This APO1 C-terminal hydrophobic domain (A1HD) interacts to form a stable dimer mainly through hydrophobic interactions within the dimer interface to create a four-stranded β-sheet positively charged surface. Structure-guided mutagenesis within this and other regions of APO1 clarified the importance of the A1HD in directing RNA and cofactor interactions, providing insights into the structural basis of selectivity on DNA or RNA substrates.

## INTRODUCTION

Nucleic acid base editing on DNA or RNA is a versatile tool that serves a variety of biological roles within living systems. The APOBEC family of proteins deaminates cytosine (C) residues on DNA or RNA into uracil, resulting in a range of biological effects such as antiviral restriction (1), immune responses (2) and aberrant mutagenesis (3,4). Within human cells, the family is divided into several subtypes: activation-induced deaminase (AID) that is a crucial part of the adaptive immune response (5,6); seven APOBEC3 proteins (A3s) that are crucial part of the innate immune system (7–10); APOBEC2 that is known to function in cardiac and skeletal muscle development (11,12); APOBEC4 whose function is still unknown (13); and APOBEC1 (APO1) (14,15), the namesake of the family and original founding member, which was initially discovered for its important role in cholesterol maintenance and lipid metabolism. Unregulated APO1 deamination activity or its deamination signature has been associated with cancer (16–20).

Despite the diverse biological function of different APOBEC family members, they all share a highly conserved core deaminase domain (CDD) structure that is composed of a five-β-stranded (β1–β5) sheet adjoined by six helices (h1–h6), with a Zn atom coordinated at the active center (21,22). Most proteins within the APOBEC family can deaminate DNA. Cytosine editing of RNA is less common, and thus far has only been shown for APO1, A3A and A3G (15,23–25). Highly specific RNA editing by APO1 was originally discovered for a single RNA base of apolipoprotein B mRNA (*APOB*) responsible for producing a stop codon and shorter version of the protein in order to regulate lipid uptake and the level of low-density lipoproteins (LDLs) (14). APO1 has been shown relatively recently to edit other RNA substrates, both within protein-coding sequences (26,27) and within 3′ untranslated regions (28,29). In all cases, it is thought that editing activity is nearly absent without the presence of at least one RNA binding cofactor as part of the highly regulated 27S editosome complex (23). The classical A1CF (APOBEC1 complementation factor) (30) and the newly discovered RBM47 (RNA-binding protein 47) (31) are the two cofactors identified so far. A1CF and RBM47 can individually confer editing activity by APO1 on known RNA substrates and are important for lipid uptake and regulating LDL levels (32,33). In addition, A1CF and RBM47 are essential genes (31,32,34,35), and play a role in RNA processing (splicing and editing), liver development and kidney function, and cancer (34,36–38).

Like other members of its family, APO1 is also capable of deamination on single-stranded DNA (ssDNA) (3,39), and such deamination activity of APO1 has been linked to cancer (16,17,19,20). It is hypothesized that APO1 may have originally evolved to act primarily on ssDNA, whereas activity on RNA may be a more recent acquisition (40,41), and notably DNA deamination by APO1 requires no other cofactors for activity. Biochemical evidence suggests that APO1 can self-dimerize (42), and the dimerization regions may overlap with those necessary for RNA editing activity (43,44). However, it is not yet clear how an APO1 dimer is formed and what functional purpose dimerization may serve. It is clear that RNA deamination by APO1 under natural conditions is very tightly regulated by some complex mechanism, as evidenced by the need for an additional cofactor and specific sequence/structure requirements of the RNA substrate (45). However, a recent study found that when rat APO1 is paired with a Cas protein/guide RNA complex and used as a site-specific cytidine base editor, rampant off-target RNA editing is observed even when expressed in a cell line without detectable expression of the two known cofactors A1CF and RBM47 (46).

Despite many years of studies investigating RNA and DNA cytosine deamination by APO1, there are still many questions remaining with regard to the molecular mechanisms behind its activity and regulation, mainly due to the lack of high-resolution structural information of APO1. In order to better understand the structural basis of APO1 activity and function, we have determined a crystal structure of APO1 that reveals dimerization occurs through its uniquely folded C-terminal domain. Subsequent structure-guided mutational and functional studies using direct biochemical and cell-based assays provide new insights into the molecular basis for APO1 activity on RNA and DNA substrates as well as the functional role of its unique C-terminal domain. The findings should contribute to the further understanding of cellular functions of APO1, as well as the potential application of APO1 in developing base editors with greatly improved substrate specificity for therapeutic purposes.

## MATERIALS AND METHODS

### Generation of a structure model for guiding mutagenesis

A preliminary model for the CDD was first generated through the SWISS-MODEL web server (47–49) by threading the sequence of APO1 onto the solved crystal structure of monomeric human APOBEC3H (50) (PDB ID: 5W45). This template was chosen by the default search criteria of the program based on the final model quality. This model was then used as input into the Topology Broker (51) of the Rosetta biomolecule modeling suite, for which the CDD (up to residue P172) was held fixed in space and the C-terminus was allowed to fold *de novo* via the *ab initio* function of RosettaScripts (52,53). APO1 sequence fragments of lengths 3 and 9 were generated via the Robetta fragment server (54). The full script and flags used are available in Supplementary File S1. Two runs of 1000 structures each were combined, and the resulting decoys were clustered via the included Rosetta.cluster software for analysis. The first run of the lowest energy cluster was used as the template for designing mutations.

### Mutation design and cloning

Mutations were chosen based on a broad-species protein BLAST by searching the RefSeq database with the human APOBEC1 amino acid sequence as a search query. At least 500 homologs were requested with the BLOSUM80 methodology. Weakly conserved hydrophobic residues on the surface of the modeled APO1 were the principal target. The initial wild-type (WT) APO1 construct was cloned into a pMAL-C5X vector. Mutations were made using Primestar Max (Takara Biosciences) high-fidelity DNA polymerase, with primer design done via Snapgene Viewer. MBP–A1CF was generated by purchasing a dsDNA gene string from Thermo Fisher (GeneArt) that contained the first 582 residues of A1CF and two phosphoserine analog mutations (S154D, S369D) thought to confer additional APO1 complementation activity (55), followed by subsequent replacement of the APO1 gene in the original vector via In-Fusion cloning (Takara Biosciences). An HRV protease site was inserted via mutagenesis into the linker region between the MBP and A1CF domains in order to allow for removal of the fusion protein, and a stop codon at the 392 positions of A1CF was later added to produce A1CF protein containing residues 1–391 experiments within this report. The reporter and editor constructs were derived from a previous study (33). All cloning was done into chemically competent Stellar (Takara Biosciences), TOP10 or DH5α cells. Plasmid mutations were confirmed with Sanger sequencing (Genewiz) and transformed into either BL21(DE3) or XA90 cells for protein expression. In some cases, the vectors showed leaky expression that made cloning difficult. To reduce the leaky expression before induction, lactose-free non-inducing plates containing ampicillin were used for selection. All final plasmid and primer sequences are available upon request.

### Protein expression and purification

Generally, 4 l of LB–ampicillin media were inoculated with starter culture and grown at 37°C until log phase; induction of protein expression was done with 100 μM final concentration of IPTG overnight at 16°C. Cells were lysed using a shear force fluid homogenizer (Microfluidics, Inc.) in a lysis buffer containing 20 mM HEPES, pH 7.5, 500 mM NaCl and 0.5 mM TCEP, with additional 100 μg/ml of RNAse A (Qiagen) added. Centrifuged cell lysates were passed over 10 ml of amylose affinity resin and washed once with lysis buffer, once with high-salt buffer (20 mM HEPES, pH 7.5, 1 M NaCl, 0.5 mM TCEP) and again with lysis buffer. Lysis buffer with 40 mM maltose was used to elute the MBP fusion proteins, and the protein was concentrated using centrifugal spin concentrators (Millipore). Concentrated MBP–APO1 proteins were further purified by size exclusion chromatography (SEC) in lysis buffer to purify the different oligomeric species. For MBP–A1CF fusion, the concentrated protein was first cleaved overnight with HRV protease at 4°C and then diluted at least 10-fold into CEX buffer A (10 mM PIPES, pH 6.5, 50 mM NaCl). Cation-exchange chromatography was performed to separate A1CF from cleaved MBP on a 6-ml Resource S column (GE) using a gradient from buffer A to CEX buffer B (10 mM PIPES, pH 6.5, 500 mM NaCl). Another SEC run was performed to further purify A1CF in a storage buffer containing 20 mM HEPES, pH 7.5, 250 mM NaCl and 0.5 mM TCEP. For more precise measurement of molecular weights (MWs), multi-angle light scattering (MALS) was performed.

### X-ray data collection and structural determination

Crystals of MBP–APO1 mXT construct were grown from a stock protein solution at 15 mg/ml within the Natrix #26 condition at 18°C. An ideal buffer condition was optimized to a final solution of 0.2 M potassium chloride, 0.2 M magnesium acetate tetrahydrate, 0.05 M sodium cacodylate, pH 6.0, and 8.5% PEG 8000. Crystals first appeared within 4 h, with a maximum size achieved after 6–7 days. Cryoprotectant was in a solution of 0.22 M potassium chloride, 0.22 M magnesium acetate, 0.05 M sodium cacodylate, pH 6.0, 9.3% PEG 8000, 20% maltose and 0.5 mM TCEP. The included 20% maltose acted as the cryoprotectant. Data were collected at an oscillation of 1° for 180° total at an optimized wavelength of 1.28345 Å. Detector distance was 472 mm, with each frame collected for 1 s. Resulting diffraction data were indexed and scaled to 3.5 Å within the *P*2_1_2_1_2 space group via HKL2000 (56).

An initial attempt of molecular replacement (MR) was performed in Phaser within the Phenix software suite (57) by searching for the MBP group separately first (PDB ID: 1ANF) (58) with a truncated form of APO2 (2NYT) (21) used for the core APOBEC domain. We eventually succeeded in using one MBP molecule as the search model to obtain a final MR solution containing eight molecules of MBP–APO2 in one asymmetric unit (ASU). The initial phases from the MR model were then improved by combining with the phases obtained from the zinc anomalous signal. Iterative model building and refinement were performed using Phenix.refine and COOT (59) to reach the final model with good statistics that are on par with the structures at a similar resolution range in the database (Table 1).

**Table 1.** Data collection and refinement statistics of APO1

Space group	*P*2_1_2_1_2
Cell dimensions	
** ** *a*, *b*, *c* (Å)	177.72, 179.21, 210.51
** ** *α*, *β*, *γ* (°)	90.0, 90.0, 90.0
Molecules/ASU	8
Resolution (Å)	50–3.50 (3.56–3.50)^a^
*R*_merge_	0.192 (0.938)
*I*/*σ*_*I*_	5.22 (1.15)
Completeness (%)	99.20 (98.10)
Redundancy	4.9 (3.7)
Refinement	
*R*_work_/*R*_free_	27.62/29.80
No. of atoms	37 624
Protein	37 254
Ligand/ion	370
*B*-factors	
Protein	136.54
Ligand/ion	142.5
rms deviations	
** **Bond lengths (Å)	0.004
** **Bond angles (°)	0.893

^a^Highest resolution shell is shown in parentheses.

### Rifampicin resistance mutagenesis assay

pMAL vectors encoding MBP fusions of each of the tested mutants were transformed into a *ung^−^* variant of NR9404 *Escherichia coli* cells, and the cells were grown on non-inducing minimal media plate overnight. Individual colonies were picked and grown in 5 ml cultures of LB media in the presence of 1 mM IPTG and 100 μg/ml carbenicillin overnight for at least 20 h, and the OD_600_ was measured and normalized to a value of ∼1.50. One hundred microliters of cells were then plated on either an LB plate containing 100 μg/ml rifampicin or an antibiotic-free equivalent after diluting the cells by 10^7^-fold. Plates were incubated at 37°C for 20 h before cells were counted. For statistical tests, data were assessed for outliers using the *R*_OUT_ nonlinear regression-based methodology at a 1% *Q* value, which resulted in the removal of no more than two outliers per assessment. The cleaned dataset was then analyzed using ANOVA with a Tukey post-test to assess the significance of different results.

### DNA deamination reactions

All DNA reactions were completed using an abasic hydrolysis technique (4,50) on a 50-nt 5′ FAM-labeled substrate with the sequence ATTAT TATTA TTCAA ATTTA TTTAT TTATT TATGG TGTTT GGTGT GGTTG. Ten-microliter deamination reactions were completed in a buffer containing 20 mM HEPES, pH 7.5, 50 mM final of NaCl, 5 mM DTT, 1 mg/ml of RNAse A and 300 nM FAM-labeled substrate at 37°C for 1 h, followed by a protein denaturation step at 95°C for 10 min and addition of 2.5 U of uracil DNA glycosylase (NEB) with incubation at 37°C for another hour to remove any uracil bases. At least 150 mM final of NaOH was added with incubation at 95°C for 10 min to induce hydrolysis of the abasic sites, and the resulting reaction mixture was run on a 20% acrylamide urea denaturing gel. The final gels were imaged with a GE Typhoon fluorescence scanner, and the percent editing of the sum of both bands was calculated for each lane. Protein concentrations used are listed, and when completed in triplicate, were done at 7 μM protein concentration for 1 h.

### RNA *in vitro* transcription

Recombinant T7 RNA polymerase (McLAB) was used to generate a 55-nt *APOB*-derived RNA substrate for additional biochemistry. ssDNA oligos were ordered and annealed together that contained the T7 promoter and desired final *APOB* RNA sequence of GGAUAUAUGAUACAAUUUGAUCAGUAUAUUAAAGAUAGUUAUGAUUUACAUGAUU. Reactions were 10–15 ml in a buffer of 40 mM Tris–HCl, pH 7.9, 6 mM MgCl_2_, 20 mM DTT, 2 mM spermidine, 500 μM dNTPs, 1 U/μl T7 RNA polymerase and 0.2 U/μl RNAseOUT (Thermo Fisher), and allowed to react overnight at 37°C. Resulting RNA was concentrated by ethanol precipitation followed by purification using TRIzol (Thermo Fisher) with the standard technique.

### RNA deamination: *in vitro* poisoned primer extension

An assay for RNA deamination was adapted for use with recombinant proteins from the poisoned primer extension design previously proposed (60). Purified recombinant MBP–APO1 and A1CF (residues 1–391) were combined at a 1:1 ratio and allowed to react on the *in vitro* transcribed *APOB* RNA described earlier. The reaction buffer consisted of 20 mM HEPES, pH 7.5, 50 mM NaCl, 5 mM DTT, 1 U/μl of RNAseOUT (Thermo Fisher) and 1 μM RNA. Ten-microliter reactions were allowed to incubate at 37°C for 1 h followed by the addition of 1.2 μM final of a 5′-FAM-labeled primer that binds downstream of the editing site with the sequence 5′-AAT CAT GTA AAT CAT AAC TAT CTT TAA TAT ACT GA-3′ and a denaturation step of 95°C for 10 min and subsequent step down to room temperature that stops the reaction, denatures the RNA strand and allows the primer to anneal. A reverse transcription buffer was then added that results in a final concentration of 2.5 U/μl Protoscript II (NEB), 1× manufacturer-recommended reverse transcription buffer, 5 mM DTT, 250 μM dTTP, dCTP and dATP, and 250 mM ddGTP (Tri-link Bio), and 0.1 U/μl of RNAseOUT (Thermo Fisher). After reverse transcription, the resulting products were run on a 20% acrylamide urea denaturing gel similar to DNA deamination.

### DNA and RNA binding assay by EMSA

Electrophoresis mobility shift assay (EMSA) was used to estimate nucleic acid binding by APO1 mutants. All EMSA gels were run on 8% acrylamide TBE native gels. The listed protein concentrations were allowed to incubate with 50 nM of either ssDNA or the *APOB* RNA in the presence of 20 mM HEPES, pH 7.5, 5 mM DTT, 10% glycerol, 50 mM NaCl and, if RNA, 0.4 U of RNAse inhibitor. The same FAM-labeled ssDNA substrate and the FAM-labeled *APOB* RNA used for deamination were used for this binding assay. Samples were incubated on ice for 10 min prior to running EMSA at 4°C in 1% TBE. Resulting quantification was done by determining the ratio of remaining unshifted DNA to the blank for each protein concentration and plotting the resulting amount of shifted DNA to the concentrations used. Nonlinear regression based on a modified Hill saturation curve (61) was determined using GraphPad Prism 8 based on three replicate experiments in order to determine 95% confidence intervals for the *K*_d_ estimates.

### RNA deamination: fluorescence localization

Fluorescence localization assays were performed as previously described (33). Briefly, the reporter and editor constructs were co-transfected into HEK 293T cells using X-TREME gene 9 transfection reagent (Sigma) using a 10:1 mass ratio excess of editor to reporter plasmid. Cells were allowed to express for 48 h, and then stained with a 5 μg/ml solution of Hoechst 33342 solution to demarcate the nuclei. Cells were imaged in an imaging buffer of 140 mM NaCl, 2.5 mM KCl, 1.8 mM CaCl_2_, 1.0 mM MgCl_2_, 20 mM HEPES, pH 7.4, and 5 mM glucose. Visualization of fluorescence was done on a Zeiss LSM-700 inverted confocal microscope using a 40× water immersion objective with a laser intensity of 15–20% and gain set to around 500 units. Excitation wavelengths for Hoechst 33342, eGFP and mCherry were set to 405, 488 and 555 nm, respectively; emission bandpass filters were set to 400–480, 490–555 and 555–700 nm. Images were captured as multichannel 16-bit grayscale intensity images 1012 × 1012 pixels across using two-line averaging and pixel dwell time of 0.8 μs. Resulting fluorescence images were quantified using the LSMtoolbox plugin of FIJI (62) by comparing the average eGFP intensity value of the observed nucleic region to the cytosolic region of individual cells, for a total of 42 cells for each trial. These values were then analyzed with Prism 8.1 using ANOVA and a Bonferroni post-test with adjustment for multiple comparisons. Resulting *P*-values were reported as *P < 0.05, **P < 0.01, ***P < 0.001 and ****P < 0.0001.

### Immunoprecipitation and western blots

For visualization of the resulting protein expression, western blots were performed by first incubating with a mouse monoclonal IgG1 anti-FLAG antibody for APO1 (Sigma), mouse monoclonal IgG1 anti-HA antibody for A1CF or mouse monoclonal IgG2a anti-α-Tubulin antibody (Genetex) as a loading control. The secondary antibody used was a cy3-labeled ECL plex goat anti-mouse IgG (GE Healthcare). All antibodies were diluted 1:3000 into PBST + 5% milk for usage. Co-immunoprecipitation on RNase A (100 μg/ml) treated cell lysates expressing only the tested APO1/A1CF co-expression vectors was completed using a classic magnetic bead co-IP kit (Pierce) as per manufacturer’s instructions, using the anti-FLAG antibody as the pull-down target. The subsequent pull-down samples were then stained with the same western antibodies as described.

## RESULTS

### Engineering a soluble APO1 protein for structural determination

**Figure 1.**
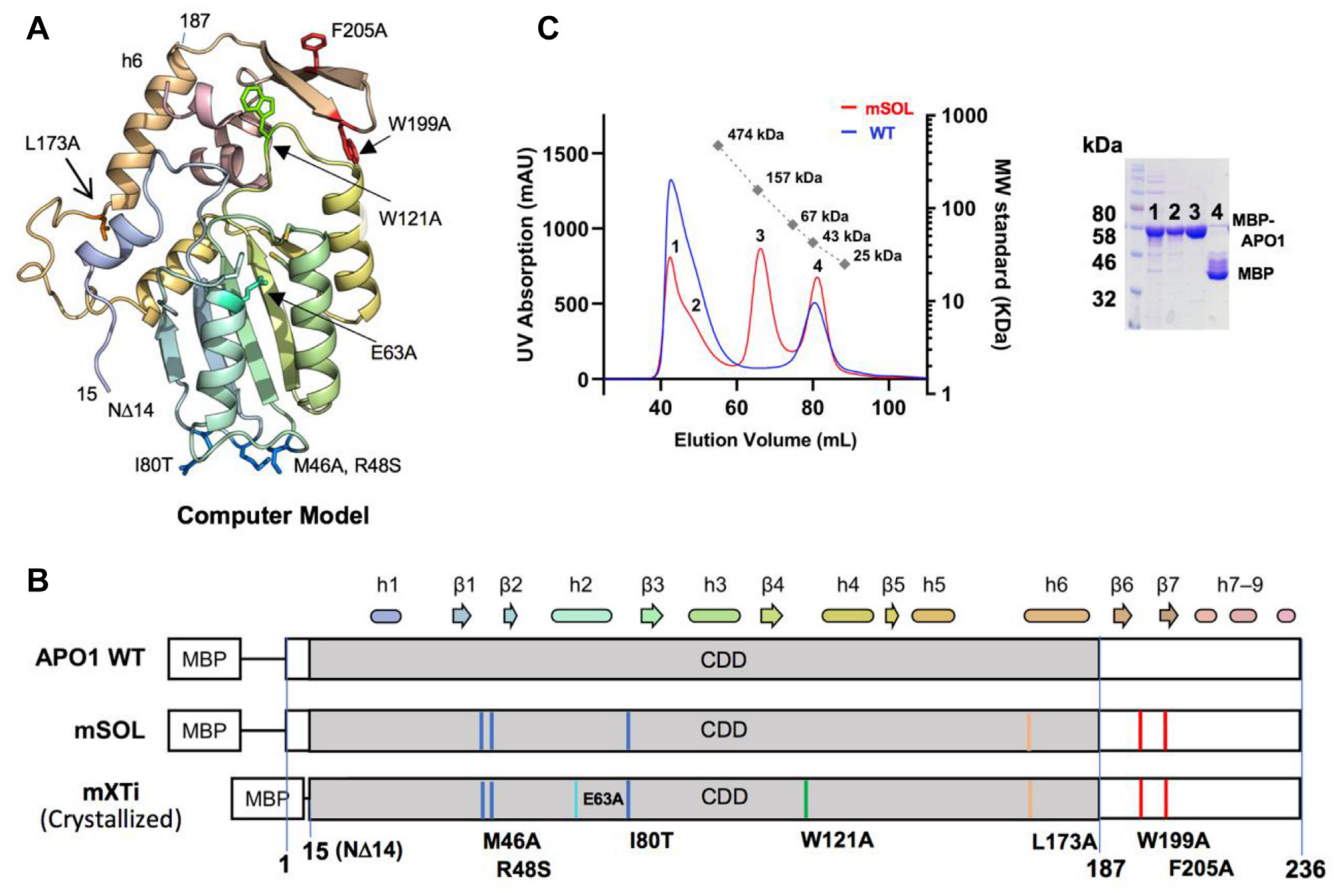
Engineering a soluble homogeneous species of MBP–APO1. (**A**) Computational modeling was used to guide mutagenesis to generate soluble APO1 for structural studies. The resulting model predicted a typical structure of the APOBEC CDD for the N-terminal residues 15–187 ending with helix 6 (h6) and a well-folded C-terminal domain containing a β-hairpin and three additional small helical domains. (**B**) Depiction of the mutations necessary to create a homogeneous species of APO1, with the predicted secondary structure elements shown to scale above the bar. The first well-behaved construct (mSOL) appeared dimeric in solution on SEC as shown in (**C**). The construct mXTi was built on mSOL with three additional mutations: W121A, deleting 14 residues of the N-terminus (NΔ14), and a catalytically inactive E63A mutation, which improved overall stability and yielded quality crystals. This mXTi construct is referred to as mXT when the catalytic residue E63 is not mutated. The locations of the mutated residues in mXTi on the structural model are indicated in (**A**). (**C**) SEC assay showing *A*_280_ traces for WT MBP–APO1 and the mSOL construct. The mSOL protein (red line) showed four peaks, with peak 1 being in the void volume and an adjacent small shoulder peak 2 containing aggregated multimeric MBP–APO1 proteins, peak 3 having an apparent MW of ∼135 kDa consistent with a dimeric MBP–APO1 (see Supplementary Figure S5C for MALS result) and peak 4 containing cleaved MBP. WT APO1 (blue line) showed a void volume peak containing aggregated MBP–APO1, and a peak containing degraded MBP. Inset: SDS-PAGE analysis of the proteins from the four elution peaks of mSOL.

Recombinant WT MBP–APO1 purified as a soluble aggregate, which is similar to what was previously reported for an *E. coli* expression system (39), although literature also reports the detection of a dimeric species when using *in vitro* translation from rabbit reticulocyte lysate (42). Full-length human APO1 contains 236 residues, with the N-terminal residues 15–187 being the CDD that bears high sequence homology to other APOBECs, and the C-terminal residues 188–236 after the CDD being unique to APO1. This unique C-terminal domain contains a high number of hydrophobic residues and has been shown to be necessary for both dimerization and RNA editing (43). Because of its hydrophobic nature, this C-terminal APO1 hydrophobic domain is referred to as A1HD hereafter. The major hurdle for the structural determination of APO1 over the past is the strong tendency of its protein to form aggregates, which may be due to the high number of hydrophobic residues in the C-terminal region that are mostly (but not exclusively) located within the A1HD domain. Initial attempts to mutate the hydrophobic residues of the A1HD were unsuccessful in generating well-behaved APO1 protein. We then performed computational modeling (see the ‘Materials and Methods’ section) to produce a model for predicting surface-exposed hydrophobic residues for mutation. Comparing this computer model (Figure 1A) with a broad sequence alignment across 100 mammalian APO1 proteins, we systematically mutated less conserved hydrophobic residues that were predicted to be on the surface in an attempt to improve solubility of the enzyme while maintaining the structural integrity.

All mutant constructs were expressed as MBP fusion proteins, and six of these mutants (M46A, R48S, I80T, L173A, W199A and F205A) showed increased solubility compared to the WT, with the locations of these residues in these six constructs mapped on the structure model and the linear sequence shown in Figure 1A and B. Combining these six mutations into a single construct, mSOL, significantly increased protein solubility compared to the individual mutants. This combined mSOL protein was purified as a clean, homogeneous peak species with an MW consistent with dimeric APO1 by SEC (Figure 1C, and Supplementary Figure S1A and B). The solubility of the mSOL mutant was further improved by adding a W121A mutation and deletion of residues 1–14 (NΔ14) of APO1 to decrease the flexibility of the linker region between MBP and APO1 (Supplementary Figure S1C), which is referred to here as mXT. Finally, the mXT construct was given the catalytically dead mutation E63A to generate an inactive APO1 construct mXTi for crystallization studies (Figure 1B).

### Overall structure of APO1

**Figure 2.**
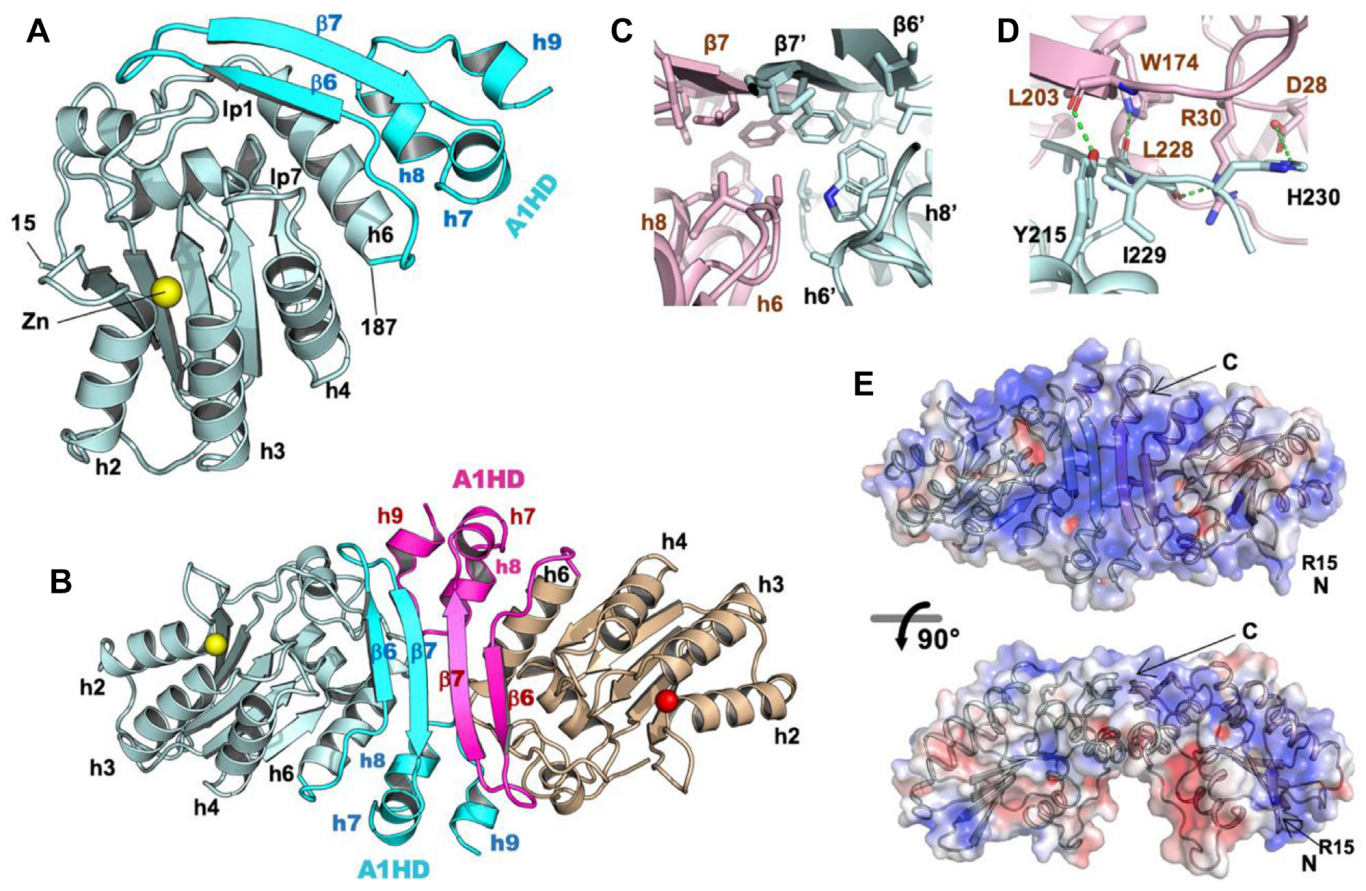
The crystal structure of APO1 reveals a well-folded C-terminal domain and a dimer formation mediated by the unique APO1 C-terminal domain (A1HD). (**A**) APO1 monomer structure, showing the arrangement of the core APOBEC deaminase domain (residues 15–187, in light blue) and its unique C-terminal A1HD (in cyan). The β-hairpin of A1HD located right after h6 of the core structure folds back toward the Zn active center and interacts with h6 and loops 1 and 7 of the core structure. (**B**) The structure of an APO1 homodimer. The dimerization is mediated through quite extensive interactions between the C-terminal A1HDs, including the paring of β7 of the two β-hairpins to form a four-stranded β-sheet, and hydrophobic packing between the two A1HD. (**C**) A view of the area with hydrophobic packing interactions between two A1HDs at the dimer interface. The interior face of both β-strands is very hydrophobic, as are the inward faces of helices 6, 8 and 9. (**D**) A view of the area with hydrogen bonding interactions on the outer edge of the dimerization interface near h8 and h9 interacting with the β7 and loop 1 regions of the opposite monomer. (**E**) Surface electrostatics generated by the APBS plugin of PYMOL 2.3 shows the β-sheet and overall protein surface is positively charged (blue coloration), forming a contiguous positively charged surface linking the N-termini at R15 and the active centers via the C-termini of each monomer.

Crystals of MBP–APO1 mXTi were obtained, and its structure was determined to the resolution of 3.5 Å in a space group of *P*2_1_2_1_2 with eight molecules per ASU (Table 1 and Supplementary Figure S2A). The structure of an individual subunit reveals that the N-terminal APO1 deaminase domain (residues 15–187) has the typical core fold of an APOBEC deaminase domain (21,22), and its C-terminal domain extension has a novel fold that is unique to APO1 (Figure 2A). The APO1 CDD structure is highly conserved with those of other APOBEC structures determined so far, as exemplified by a root-mean-square deviation of 1.01 Å for its superimposition with the structure of AID (63) (Supplementary Figure S2B). In the crystal packing of the MBP–APO1 fusion, there are only two direct contacts between APO1 molecules (Supplementary Figure S2A), one of which is minor contact with a buried area of only ∼451.2 Å^2^ with mostly hydrophilic interactions. The other contact interface is much larger, with a buried surface area of 1526.5 Å^2^ that consists of mostly hydrophobic interactions mediated by the C-terminal A1HD (Figure 2A and B). The relatively large buried hydrophobic interface area of 1526.5 Å^2^ is indicative of a true biological interaction (64,65) and is consistent with the stable dimer observed in solution.

### Structure of the C-terminal A1HD region

The A1HD is composed of a β-hairpin (β6 and β7) and three small helices (h7, h8, h9). Within an APO1 molecule, the A1HD uses its β-hairpin and h7 and h8 to form hydrophobic interactions with its own CDD, positioning its β-hairpin across the top side of the Zn active center and in close proximity to loops 1 and 7 of the CDD (Figure 2A). The well-structured A1HD and its packing with the CDD effectively shields many of the hydrophobic residues on the core domain side as well as the A1HD side. Overlapping the crystal and the modeled structures reveals that the prediction of the 3D fold of A1HD and its interactions with the CDD differed, even though the CDDs are quite similar (Supplementary Figure S2C). The overall fold of the A1HD has no close match to any other folds in the structure database, although it somewhat resembles an α–β plait domain within the list of CATH domain definitions (66).

### Dimerization interactions mediated by the A1HD

The A1HD mediates dimerization of APO1 primarily through the pairing of the two β-hairpins via β7 of the two monomers, forming a four-stranded β-sheet connecting the two subunits. The interior face of this β-sheet interacts extensively with helices h6 and h8 of A1HD through hydrophobic residues to create a hydrophobic core between the two APO1 subunits (Figure 2C). There are additional intermolecular interactions between h9 from one subunit with the N-terminal h1/loop 1 region and h7/h8 with the β-hairpin of the other subunit (Figure 2D), as well as the hydrogen bonds formed between the two anti-parallel β-strands at the interface. The structure reveals what was previously proposed to be a leucine zipper motif on h6 for dimerization of APO1 (15,42) instead forms intramolecular hydrophobic packing interactions with the A1HD of the same monomer.

Two early models for APO1 structure and dimerization had previously been proposed based on the crystal structures of free cytidine deaminases from *E. coli* (ecCDA) (67) and yeast (CDD1) (68). These modeling studies of APO1 precede the publication of the first crystal structure of an APOBEC protein revealing the characteristic fold of APOBEC core deaminase features (21), and the reported models and the dimerization modes are very different from the crystal structure shown here (Supplementary Figure S2D). However, many of the previous mutational studies to investigate the dimerization of APO1 can now be explained based on the crystal structure. Mutations of hydrophobic residues near the C-terminus such as L135F, F156L and L189F (67) were shown to disrupt dimerization likely through disrupting folding and destabilization of the structure, as these are inward-facing buried residues. Additional mutations of hydrophobic residues around this region, such as L182A and I185A, were found to disrupt RNA editing activity (43), which likely is due to the disruption of the internal hydrophobic packing between the A1HD and its CDD. Furthermore, it was also reported that C-terminal truncations of this A1HD disrupted dimerization (43), consistent with the role of A1HD in mediating the dimerization interface as observed in this structure (Figure 2B).

The dimeric structure of APO1 reveals a large positively charged surface spanning across the two paired β-hairpins of A1HDs and branching out to the two active centers near the N-termini (Figure 2E). Mutations of residues within this positively charged patch, such as R16, R17, R33 and K34, showed a significant impact on both RNA binding and editing (43,46,67,69), and this region was also shown to function as a biological nuclear localization signal (69). A C-terminal deletion of the A1HD to residue 196, which removes most of the β-hairpin structure motif and much of this positively charged surface, showed a major reduction in RNA editing activity (43). We further deleted the C-terminus to residue 188 (CΔ48) and found this deletion mutant showed diminished deaminase activity on both RNA and DNA (Supplementary Figure S3A), suggesting that the C-terminal A1HD plays a role in regulating APO1 activity on RNA and DNA deamination, possibly by preventing nonspecific aggregation resulted from the extra-hydrophobic residues at the C-terminal h6 of the CDD.

### The crystallized APO1 is active on DNA but inactive on RNA deamination

**Figure 3.**
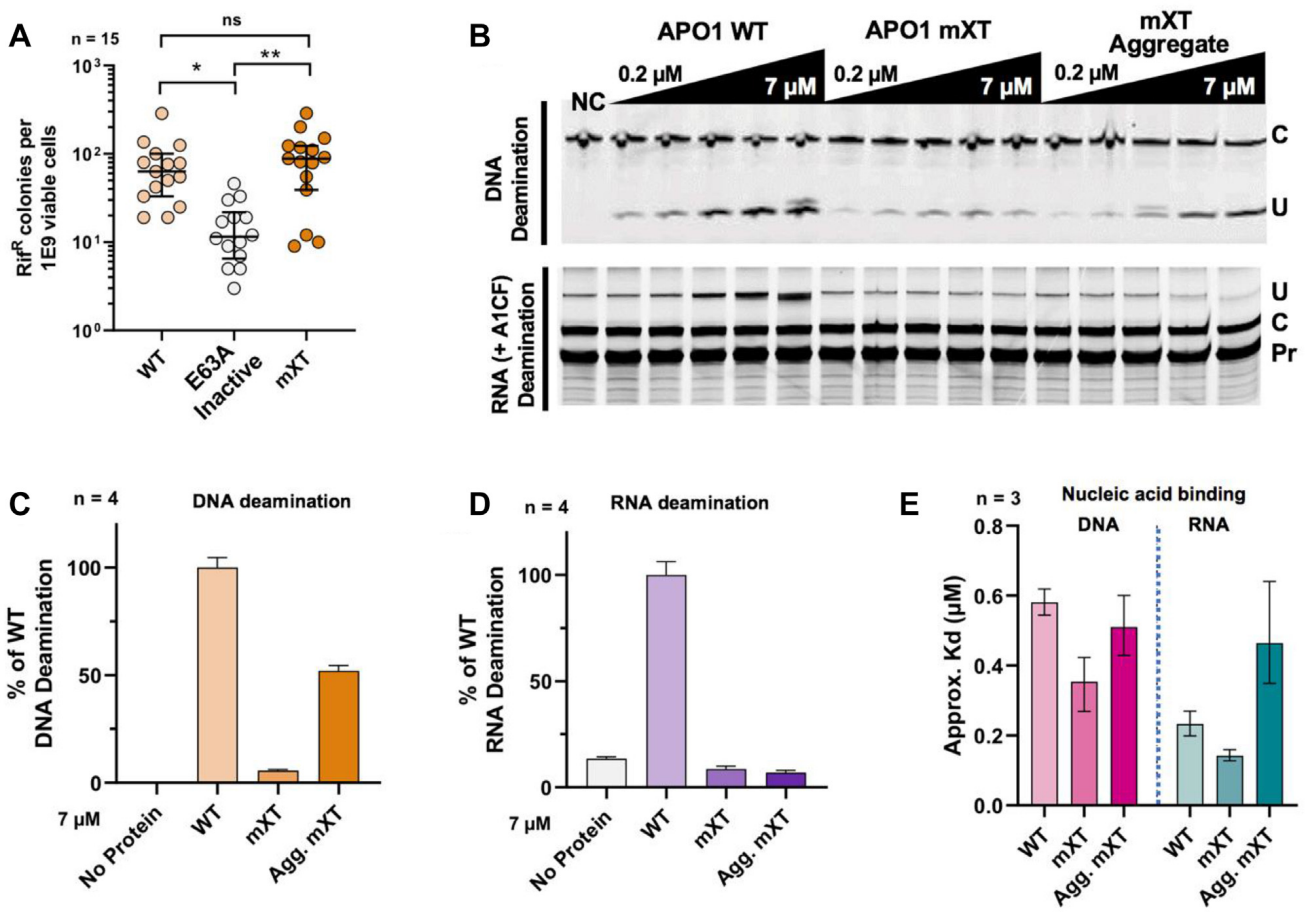
The crystallized form of APO1 has activity on ssDNA but not RNA. (**A**) Rifampicin resistance mutagenicity assay of MBP fusions for APO1 WT, an E63A inactive mutant and the crystallized mXT form of APO1. Both WT and mXT showed statistically equivalent levels of increased rifampicin resistance induction compared to the inactive control, as determined by ANOVA with Tukey post-test after ROUT outlier adjustment. (**B**) Example of gel analysis of *in vitro* ssDNA (top) and *APOB* RNA deamination (bottom) assays comparing the activities of the aggregated WT MBP–APO1 protein with the aggregated and dimeric forms of the mXT mutant protein. The top panel shows that both forms of mXT protein had an obvious reduction of DNA deaminase activity compared with WT APO1, with the dimeric mXT showing a more dramatic effect than the aggregated form. NC is a negative control. The bottom panel shows RNA deaminase assay using poison primer assay (PPA). In this PPA, poisoned primer extension uses dideoxyguanosine to halt the reverse transcription (RT) extension if there is a cytosine still (unedited in the figure), but will extend past the edited U to give a longer product at the top of the gel. (**C**) Quantification of 3× ssDNA deaminase activity assays [see panel (**B**), top] at the highest concentration tested; the aggregated mXT had ∼50% of the activity of WT on DNA, whereas the dimeric mXT had ∼5% of WT activity. (**D**) Quantification of 3× RNA deaminase activity assays [see panel (**B**), bottom] at the highest concentration tested. Neither form of mXT had any level of RNA editing greater than the background. RNA deamination experiments were done in the presence of A1CF. Error bars represent the standard deviation across three independent experiments. (**E**) Results of ssDNA and *APOB* RNA binding assay using EMSA. The results showed that, for ssDNA binding, both the dimeric and aggregated mXT proteins had a slightly stronger binding affinity than WT APO1 (left). For APOB RNA binding, however, only the dimeric mXT showed stronger binding, and the aggregated mXT showed weaker binding than WT. The plot shows the mean *K*_d_ value from three independent experiments with error bars marking 95% confidence intervals for each sample. Corresponding EMSA gels are provided in Supplementary Figure S7.

Because the crystalized APO1 construct contains several mutations (Figure 1A and B), we wanted to assess whether the construct was still catalytically active after restoring the catalytic residue E63A in mXTi back to glutamate. A rifampicin resistance mutagenesis assay showed that the MBP-mXT construct showed no significant change in activity compared to WT, whereas both had significantly higher activity than the catalytically inactive mutant E63A (Figure 3A) as determined by ANOVA with Tukey post-test for multiple comparisons. Specifically, the mXT construct showed a median of 88 Rif^R^/10^9^ viable cells, while the WT APO1 showed a median of 63 Rif^R^/10^9^ viable cells. These observations were also consistent with a previously reported mutagenicity of ∼50 Rif^R^/10^9^ viable cells for untagged human APO1, which is notably far less than the mutagenicity of APO1 from other mammals such as rat and rabbit (3,70). These results indicated that the mXT construct is still able to deaminate DNA in the bacterial rifampicin resistance assay.

The activity on DNA and RNA deamination was also assessed using purified recombinant proteins *in vitro*. Even though mXT protein can be purified as a stable dimer in addition to the aggregated species (Supplementary Figure S1C), WT APO1 protein cannot be separated as stable dimer form and only aggregated species can be obtained (Figure 1C). For comparison with the aggregated WT APO1, the aggregated mXT species, as well as the dimeric mXT, was purified for deamination assays. Results showed that the aggregated mXT showed obvious DNA deamination activity at ∼50% of WT activity (Figure 3B and C). Interestingly, dimeric mXT showed a DNA deaminase activity of ∼10% of the aggregated mXT, and only ∼5% of WT activity (Figure 3C). By comparison, both the aggregated and dimeric mXT in the presence of purified A1CF cofactor protein (Supplementary Figure S3B) showed no RNA deaminase activity on a 55-nt *APOB* RNA substrate above the background level, while the aggregated WT APO1 showed robust RNA deaminase activity in this *in vitro* primer extension assay (Figure 3B and D). These *in vitro* assays showed that the mXT construct is active in DNA deamination, but inactive in RNA deamination.

To examine whether the altered activity on DNA and RNA deamination by the purified mXT construct was related to changes in substrate binding, the same 50-nt ssDNA and 55-nt structured *APOB* RNA substrates used for deamination assays were assessed for binding to the purified APO1 proteins using EMSA (Figure 3E and Supplementary Figure S4). For DNA binding, the dimeric and aggregated forms of mXT showed slightly stronger binding than WT (left-hand side in Figure 3E), despite both forms having much reduced DNA deaminase activity (Figure 3C). For RNA binding, even though *APOB* RNA deamination by APO1 requires a cofactor (such as A1CF), WT APO1 alone showed obvious binding to *APOB* RNA (right half in Figure 3E), with a *K*_d_ value of ∼0.23 μM. The two forms of the mXT mutant, despite having lost deaminase activity on *APOB* RNA (Figure 3D), can also bind this RNA, with the dimeric mXT showing even stronger binding (0.14 μM) than WT, while the aggregated mXT showed weaker binding (0.46 μM) (Figure 3E). These results indicate that neither the reduced DNA deaminase activity nor loss of RNA deamination for the mXT mutant can be correlated to the reduction of binding affinity for the DNA or RNA substrate. It was thus possible that mutation of one or more of the mutated residues in the mXT APO1 construct may result in change of certain critical type of interactions with the ssDNA or RNA substrate, leading to the reduction of DNA deamination activity, or loss of activity on RNA deamination.

### Residues of APO1 loop 7 important for deamination

**Figure 4.**
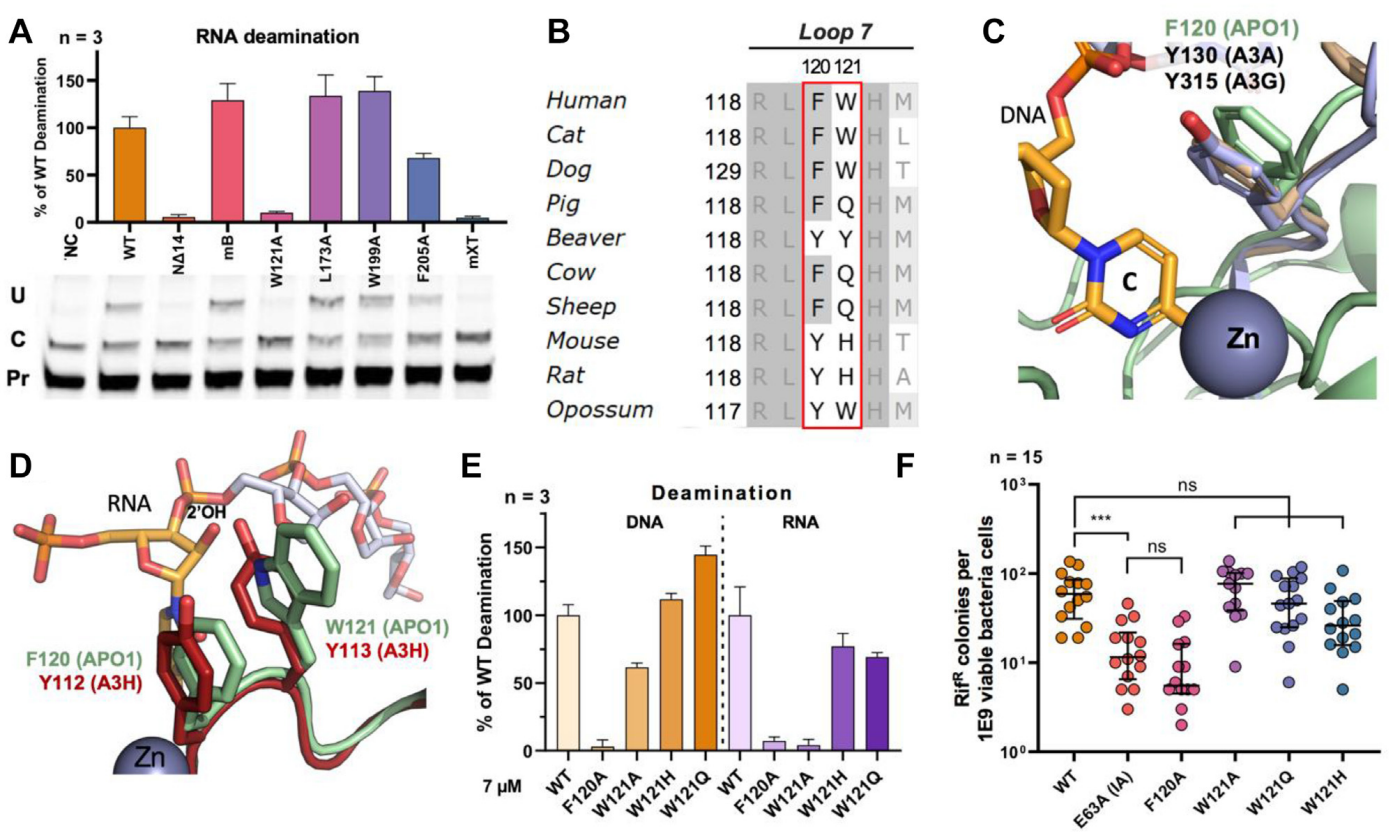
W121 on loop 7 is critical for RNA editing but not DNA deamination by APO1. (**A**) RNA deamination of APO1 mutants that contain each of the six mutations from the mXT construct using the primer extension assay. MBP–APO1 fusion proteins were purified and used for the assay for all mutants. NC denotes a negative control without APO1. The mB mutant represents the M46A, R48A and I80T mutation combination; error bars represent the standard deviation calculated from three individual deamination trials. (**B**) Sequence alignment of several mammalian APO1 suggests that F120 is highly conserved as either an aromatic phenylalanine or tyrosine, but W121 is less conserved and can be a glutamine or histidine at this location. (**C**) Structural superimposition of APO1 with several other known APOBEC–ssDNA structures. F120 of APO1 (in light green) aligns in 3D space with Y130 of A3A (PDB ID: 5KEG, in purple) and Y315 of A3G (PDB ID: 6BUX, in tan), which have both been observed to form pi-stacking interactions with the targeted cytosine in the active center. (**D**) A structural superimposition of APO1 (in light green) with A3H (PDB ID: 5Z98, shown in red) shows that APO1 W121 aligns well with A3H Y113, a residue that interacts with the 2′-hydroxyl of the ribose moiety of the RNA in the A3H–RNA complex structure. (**E**) Result of DNA and RNA deaminase activities of the mutations W121A, W121H and W121Q. W121A had ∼60% of the activity on ssDNA but showed a near-complete abolishment of activity on *APOB* RNA. W121H and W121Q both showed activity on ssDNA and RNA. F120A abolished activity on both ssDNA and RNA substrates. (**F**) A rifampicin resistance mutagenesis assay in bacterial cells confirms that F120A is catalytically inactive, whereas the W121A, W121Q and W121H mutants showed a similar level of restoration of rifampicin resistance as WT.

To further understand why the crystallized APO1 construct mXT was able to deaminate ssDNA but showed almost no activity on RNA, we generated constructs containing each of the six mutations in mXT to see whether any of the individual mutations preferentially affected RNA deamination. Two such mutant proteins purified as MBP–APO1 displayed a near-complete loss of RNA deaminase activity in the *in vitro* assay: mutants W121A and NΔ14 (N-terminal truncation of 14 residues) (Figure 4A). For the construct NΔ14, the exact reason for the loss of RNA deaminase activity is unclear, even though it was suggested from a prior report that deleting the N-terminal 15 and 30 residues of APO1 diminished A1CF cofactor binding (69). As for mutant W121A, the mutated residue W121 is located on APO1 loop 7, one of the three active site loops (loops 1, 3 and 7) known to interact with substrates in APOBEC proteins (71–76). W121 is mostly conserved among APO1 across species and can be a His or Glu in some mammals (Figure 4B). The loop 7 of A3H is shown to interact with RNA (71,72,74). While not acting as a substrate, the RNA in the A3H co-crystal structures interacts with several residues of loop 7. Overlaying the crystal structure of A3H to APO1 shows that W121 of APO1 is analogous to Y113 of A3H in 3D space (Figure 4D), where Y113 interacts with a ribose 2′-hydroxyl of the bound RNA. W121 of APO1 and the other three types of residues (Y121, H121 and Q121; Figure 4B) seen at this position across the mammalian APO1 homologs are all capable of forming similar hydrogen bonds as Y113 of A3H and may serve a comparable role in mediating RNA substrate recognition over DNA. On the other hand, APO1 residue F120 next to W121 is also very conserved and can be a Tyr (Y120) in other organisms (Figure 4B). Recent structure studies have revealed a Tyr (Y) residue at the equivalent position of APO1 F120 on loop 7 of A3A (75,76), A3BCD2 (75) and A3GCD2 (77); all form critical aromatic pi-stacking interactions with the target C for deamination (Figure 4C). As such, unlike W121, F120 in APO1 may be critical for pi-stacking with the target C for deamination on both ssDNA and RNA substrates, and mutating F120 on loop 7 is expected to abolish C deamination on both substrates.

### The role of W121 on APO1 loop 7 in substrate selection

To further investigate the relative importance of F120 and W121 in APO1 deamination of DNA and RNA, the single point mutations F120A, W121A, W121H and W121Q on loop 7 were generated to examine their DNA and RNA deamination *in vitro*. The result showed that F120A had near-complete abolishment of deaminase activity for both RNA and DNA substrates, while W121A only showed near-complete abolishment of activity on RNA but retained ∼60% of WT activity for deamination on DNA (Figure 4E). Nonetheless, unlike the W121A mutation, W121H and W121Q both displayed RNA deaminase activity, with ∼75% and ∼70% of WT activity, respectively (Figure 4D). Interestingly, both W121H and W121Q showed even stronger DNA deamination activity compared to WT (Figure 4E). A subsequent Rif^R^ assay in bacterial cells verified that F120A behaved the same as the catalytically dead E63A mutant (Figure 4F), whereas W121A, W121H and W121Q all displayed DNA deamination activity comparable to WT (Figure 4F); this effect is consistent with what was observed between WT and the mXT construct in the Rif^R^ assay (Figure 3A).

DNA and RNA binding assays showed that the four mutants (F120A, W121A, W121H and W121Q) had estimated *K*_d_ values between 0.39 and 0.75 μM (Supplementary Figure S4) and did not show an obvious correlation between the *K*_d_ value changes and the deaminase activity levels observed for these mutants. These results suggest that, although F120 and W121 on loop 7 play a role in interacting with both RNA and ssDNA substrates, residue 121 appears to be critical in differentiating RNA versus ssDNA substrates for deamination, possibly through interacting with and positioning RNA in a certain orientation, or by coordinating with a cofactor (such as A1CF or RBM47) to interact with RNA in a specific fashion needed for deamination. A more mechanistic understanding of the role of W121 in RNA deamination may require a co-crystal structure of APO1/cofactor binding to substrate RNA in the future.

### APO1 dimerization and its role in RNA editing activity

**Figure 5.**
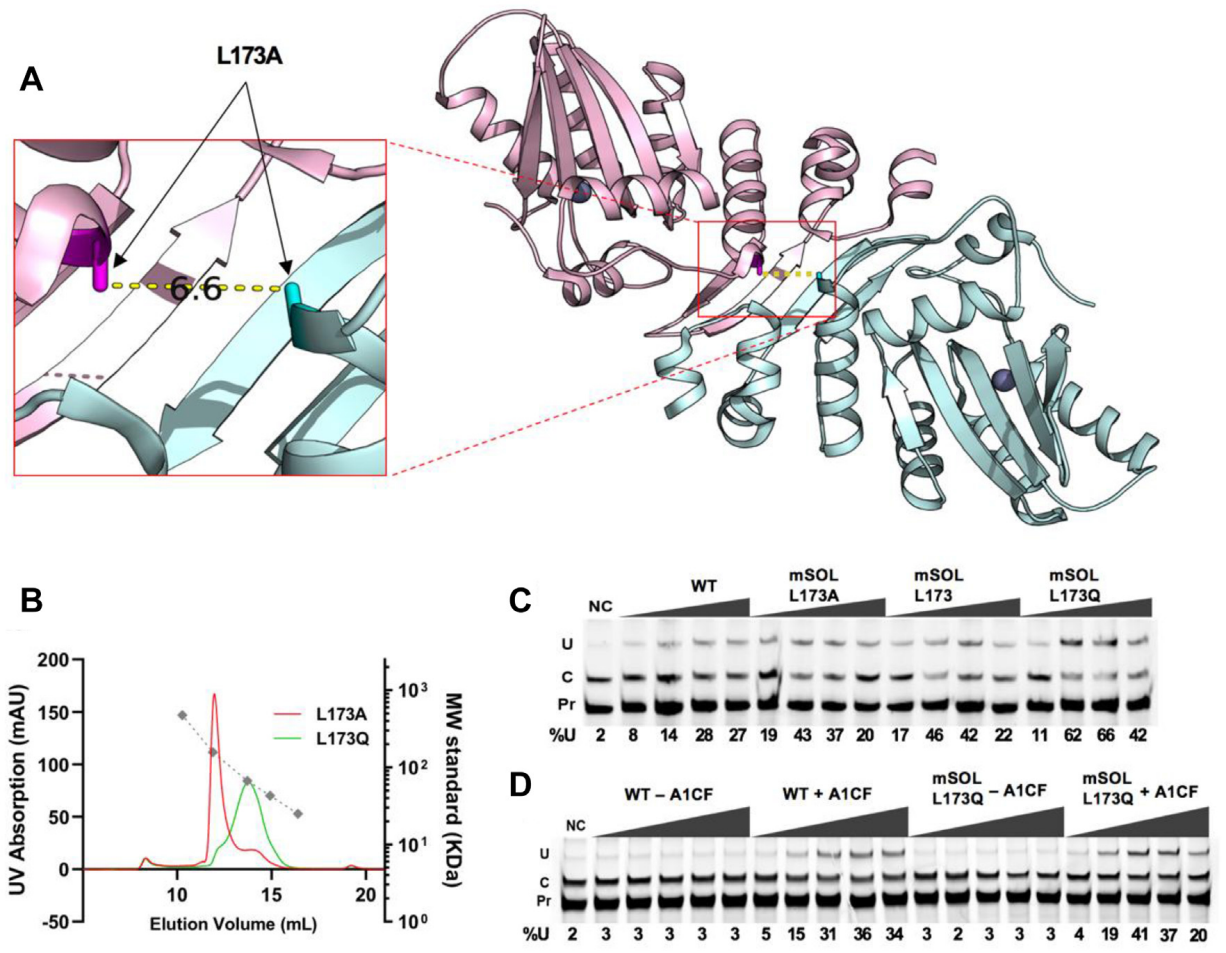
L173Q mutation of APO1 disrupts homodimerization but not activity on RNA. (**A**) Close-up view of APO1 structure at the dimer interface, showing that there is ∼6.6 Å distance between the Cβ atoms of each A173 of a dimerized pair. This distance would be able to fit two leucine residues at position 173 within the dimer interface but is not sufficient to fit the larger polar residue glutamine without causing steric hindrance and disrupting the dimer interactions. (**B**) SEC assay profile showing that an L173Q mutation on the dimeric MBP–APO1 mSOL construct resulted in shifting the dimeric elution peak position of L173A to a position with smaller apparent MW consistent with a monomeric form. Subsequent MALS confirmed that the L173Q mutant is monomeric with an MW of 67 kDa (see Supplementary Figure S5C). (**C**) RNA deamination comparing WT MBP–APO1 with the mSOL construct carrying L173A, L173 (mutated back to L as in the WT) or the monomer mutant L173Q. All show comparable levels of RNA editing in the presence of A1CF. (**D**) WT and monomeric mSOL L173Q were assayed for RNA editing activity with and without the presence of A1CF. Both WT and the L173Q construct showed comparable RNA editing activity in the presence of A1CF and no activity in the absence of A1CF. NC denotes a no-APO1 negative control in panels (**C**) and (**D**).

While the dimer form of APO1 is consistent with the prior biochemistry evidence (15,42–44), there is a possibility that mutations of the crystallized construct may promote the dimer formation. The mapped locations of the eight mutations on the dimer structure (Supplementary Figure S5A) indicate that only L173A is buried inside the dimer interface, with other residues exposed to solvent. The L173A mutation is located right at the apex within the dimer interface on h6, where four helices meet (Figure 5A). The two small alanine residues at position 173 are ∼7 Å apart in the dimer structure. This dimer structure predicts that the 7 Å distance can fit two WT leucine residues well at 173 position (L173; Supplementary Figure S5B) buried inside the dimer interface (Figure 5A). On the contrary, if position 173 is replaced with a larger polar residue glutamine (Q173), the structure predicts clash between two Q173 residues at the dimer interface and disruption of the dimer into monomer form. To verify this structural prediction about the effect of residue 173 on dimerization, we purified APO1 mutant proteins with position 173 being Leu (173L, WT), Glu (173Q) or Ala (173A) in the context of mXT and analyzed their oligomeric status using SEC and MALS (Figure 5B and Supplementary Figure S5C). The result showed that, while the mXT construct (with 173A) was a stable clean dimer with an apparent MW of 138 kDa (calculated MW to be 134 kDa for a dimer), the mXT-173Q construct showed a clean monomeric form with an apparent MW of 66 kDa (Figure 5B and Supplementary Figure S5C), consistent with the prediction that this mutation can disrupt the dimerization interface. There is a tiny dimer peak (∼1–2%) for mXT-173Q. The mXT-173L construct also yielded a protein that formed a dimer peak similar to that of the 173A mutant, although this mXT-173L construct was far more prone to aggregation. These results, taken together, confirm the dimeric interface observed in the crystal structure mediates stable dimer formation and that substitution of the residue 173 within the interface by a larger polar residue (173Q) is sufficient to disrupt the dimer into a monomer.

We next examined the role of dimerization in RNA editing by comparing RNA deamination in the presence of these same mutations (L173L, L173A or L173Q), on the mSOL construct (Supplementary Figure S1A). Both the dimeric L173A and monomeric L173Q construct showed deaminase activity comparable to WT in the presence of A1CF cofactor (Figure 5C), suggesting that APO1 dimerization per se is not needed for deamination activity on *APOB* RNA. One caveat is that the activity of L173Q could come from the residual dimer, even though such possibility is low as only the fractions centered around the monomeric peak were used for the activity assay. This result is consistent with a recent report where GFP fused to the C-terminus (APO1–GFP) but not to the N-terminus (GFP–APO1) significantly lowered dimerization, and this APO1–GFP fusion defective in dimerization is still active (44). To test whether APO1 dimerization is potentially a mechanism for inhibiting its unregulated RNA editing in the absence of the cofactor A1CF, we tested the activity of the monomer L173Q mutant on *APOB* RNA with and without the presence of A1CF (Figure 5D). This monomer mutant L173Q showed RNA deaminase activity only in the presence of A1CF, similar to the WT control, suggesting that prevention of rampant RNA editing in the absence of a cofactor is not the biological function of APO1 dimerization. Interestingly, it was reported that residues 173–182 on h6 function as a nuclear export signal (NES) (69). Residues 173–182 are essentially hydrophobic and mostly masked by the C-terminal A1HD domain of the same molecule in the dimer form. Thus, for this NES sequence to mediate nuclear export of APO1, conformational changes of the dimer form will be needed to expose this hydrophobic 12-amino acid NES, which could be achieved by interacting with its cofactor and/or RNA to alter the conformation of A1HD.

### Assessing APO1 activity with a cell-based RNA editing assay

**Figure 6.**
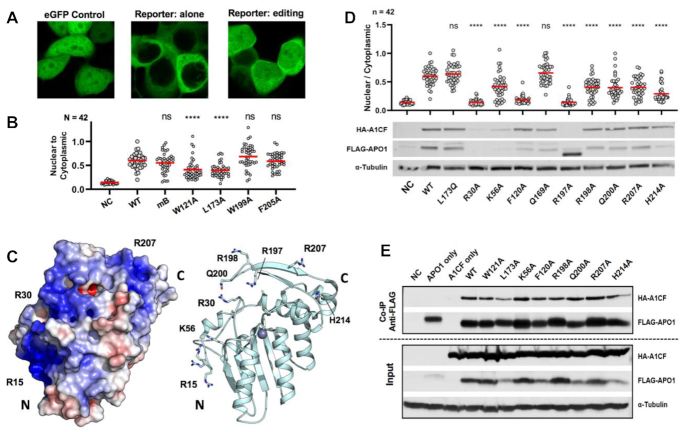
RNA editing activity of various mutants of APO1, as shown by a cell-based fluorescence re-localization assay. (**A**) Confocal microscopy images showing the cell-based RNA editing assay by measuring fluorescence localization in 293T cells expressing an eGFP reporter and APO1/A1CF editor (see Supplementary Figure S6A and B). Unmodified eGFP is uniformly distributed throughout the cytosol and nucleus (left). The eGFP–NES reporter without RNA editing is localized only in the cytosol (middle); RNA editing by the co-expressed APO1/A1CF editor generates a stop codon before the NES in the mRNA encoding eGFP–NES so that the resulting eGFP localizes to the nucleus (right). (**B**) Cell-based RNA editing assay of four mutants derived from the original mXT construct. The mB construct, W199A and F205A showed no change in RNA editing compared to WT. L173A and W121A both showed significantly reduced RNA editing compared with WT. Significance is shown relative to WT (****P < 0.0001; ns denotes *P* > 0.05) as calculated by ANOVA assessment and Bonferroni post-test. Additional comparisons are shown in Supplementary Figure S6C. NC is a negative control where only mCherry was expressed. (**C**) Location of mutations made across the loop 1 and A1HD regions of APO1 within the positively charged surface (left). (**D**) Cell-based RNA editing assay of the mutants within the positively charged surface area. The L173Q monomer mutant showed WT level activity, whereas R30A, F120A and R197A all showed complete abolishment of activity. The bottom panel is a western blot for each mutant. R30A had no detectable protein, and R197A had protein bands that appear degraded. (**E**) Co-immunoprecipitation of A1CF with some of the FLAG–APO1 mutants from this study using anti-FLAG antibody from cell lysates suggests none of the mutations dramatically affected A1CF binding.

To validate the RNA deamination activity seen with the *in vitro* poisoned primer extension and further characterize the RNA editing activity of APO1 mutations in a cellular environment, we used an in-cell fluorescence assay to detect and quantify RNA editing activity as previously described (33). This fluorescence assay can detect editing of a fraction of mRNA molecules of a reporter protein by visualizing/quantifying the fluorescent shift into the nucleus from the cytosol. This is achieved by inserting the targeted RNA sequence between eGFP and MAPKK NES (Supplementary Figure S6A), which will express eGFP signals only in the cytosol, leaving the nucleus free of fluorescent signals. Editing of the targeted C to U on the mRNA creates an early stop codon before the NES, resulting in a quantifiable shift of eGFP fluorescence from the cytosol to the nucleus (Figure 6A), which can be used as a sensitive reporter for RNA editing by APO1/cofactor expressed from an editor construct in a cellular environment. The RNA region used here for this cell-based assay is a 27-nt segment of human *APOB* RNA that has been shown to be specifically edited by APO1 when paired with A1CF (or RBM47) in previous reports (33,45,78). The editor construct expresses APO1, A1CF and mCherry from the same open reading frame of a single mRNA, but a 2A peptide (79,80) is inserted between each protein to allow the generation of individual proteins during translation (Supplementary Figure S6A). This design enables the visualization of mCherry expression encoded by the very 3′-end of the mRNA as a reliable indicator for the expression of the upstream APO1 and A1CF within the same cell, allowing for easy normalization across a high number of quantified cells, resulting in a robust and reproducible measurement for RNA editing in a cellular environment.

Among the six APO1 constructs tested for RNA editing in this cell-based fluorescence shift assay, mutants mB (M46A–R48S–I80T), W199A and F205A all had RNA editing activities comparable to WT (Figure 6B), consistent with the result obtained using *in vitro* primer extension (Figure 4A). However, W121A was significantly reduced compared to WT (Figure 6B), implying this mutation may not be a complete knockout of activity on RNA as observed with *in vitro* primer extension (Figure 4A). L173A also showed lower activity than WT, unlike the comparable activity seen with primer extension (Figure 4A). The differences in the RNA editing activities observed for W121A and L173A may reflect the differences between the two assay systems. While we do not have a good understanding for these differences, the cell-based fluorescence assay appears to have a much higher dynamic range in detectable editing on each of the many cells measured in each independent study and may be sensitive enough to detect weak activity fluctuations that are not seen with primer extension.

### Effects of positively charged surface mutations on RNA editing

The APO1 molecular surface is very basic and contains a continuous patch of positively charged and polar residues (Figure 6C, left), starting from the N-terminal (labeled N) residues R15/R16/R17 to the C-terminal (labeled C) area around R207/H214 of the A1HD (Figure 6C, right). Dimerization via A1HD connects and widens the positively charged surface (Figure 2E). It has previously been shown that mutations of some of these positively charged residues, such as R33A, K34A or at least two of the residues R15/R16/R17, can dramatically reduce RNA editing activity and disrupt binding to A1CF (46,67,69). To further understand the potential role of this extensive positively charged surface, selected charged/polar residues within this surface area (Figure 6C, right) were mutated to alanine and assessed for RNA editing activity using the cell-based fluorescence localization assay described above. The previously tested mutations F120A and L173Q were also included as inactive and fully active control mutants.

The RNA editing result showed that mutants R30A and R197A, like the inactive control F120A mutant and the mCherry only negative control, showed no RNA editing activity (Figure 6D, and Supplementary Figure S6B and C). The western blot analysis of APO1 and A1CF protein expression in cells showed that the mutant R30A had no detectable APO1 protein, and R197A had a truncated form of APO1 with essentially no detectable A1CF (Figure 6D). Because mCherry expression is a reliable marker for expression of upstream APO1 and A1CF in the editor construct (Supplementary Figure S6A), mCherry fluorescence for these samples (Supplementary Figure S6B) suggests that the R30A and R197A APO1 mutants were expressed, but post-translational destabilization or degradation may have occurred for these mutant proteins. Therefore, the absence of R30A protein or degradation of R197A protein may explain the lack of observed editing activity for these two mutants. Five other mutants, K56A, R198A, Q200A, R207A and H214A, showed significantly reduced RNA editing when compared to WT APO1 (Figure 6D), and H214A had the strongest effect. For comparison, the mutation Q169A that was purposely picked from outside the positively charged surface, together with dimer disrupting monomer mutant L173Q, showed full WT editing activity. These mutational results, together with the previously reported mutational studies (46,67,69), suggest that these positive/polar residues on the positively charged surface of APO1 play an important role in mediating RNA editing.

To investigate whether the reduced RNA editing of these mutations was due to reduced APO1–A1CF binding, co-immunoprecipitation was performed using the FLAG-tagged APO1 that is co-expressed with A1CF in HEK 293T cells. The result showed that, except for L173A, which had slightly reduced A1CF binding, all other tested mutants had WT levels of A1CF binding, including the F120A and W121A (Figure 6E). This result suggests that the reduced RNA editing activity for these APO1 mutants is not correlated with the reduction of A1CF binding.

## DISCUSSION

Here, we have described the crystal structure of APO1 and its structure-based functional characterization. The APO1 structure reveals an N-terminal CDD that is similar to the canonical APOBEC deaminase domain and an extra well-structured C-terminal A1HD domain with a unique fold (Figure 2A). The A1HD interacts extensively with its counterpart from another APO1 monomer through hydrophobic packing to form a stable dimer via 2-fold rotational symmetry (Figure 2B), which can be disrupted by a steric point mutation at the interface. The surface of this paired dimer creates a four-stranded β-plane between the two A1HDs that connect the highly positively charged surface of the two subunits (Figure 2E).

The crystallized APO1 mutant had deaminase activity on DNA but not on the *APOB*-derived RNA that is known to be specifically deaminated by WT APO1 (Figure 3A–D). W121A was identified as the key mutation that greatly impaired deaminase activity on RNA but not on DNA (Figure 4A). W121 of APO1 is on loop 7, a region known to be involved in substrate specificity in other APOBECs (71–75). The observed differential effect of W121A on RNA versus DNA deaminase activity suggests that residue 121 may perform a role in selectively targeting RNA substrates for deamination. Interestingly, mutants W121Q and W121H did not abolish RNA deamination (Figure 4E), suggesting that the function of this residue possibly involves the formation of a hydrogen bond (Figure 4D), which can be fulfilled by not only tryptophan (W121), but also glutamine (W121Q) or histidine (W121H), but not alanine as in W121A. We noticed that, while no RNA deamination was detectable for W121A mutant in the primer extension assay *in vitro*, low-level activity was detected when using a more sensitive cell-based reporter assay (Figure 6B). This suggests that there may be a continuum effect in determining substrate specificity.

In addition to loop 7, residues on loop 1 and the N-terminus of APO1 also have previously been shown to have an effect on RNA deamination activity (46,67,69), and these residues are located on the positively charged surface running from the N-terminus to the C-terminal A1HD (Figures 2E and 6C). Using the cell-based RNA editing assay, we show here that additional mutations within this positively charged region on the A1HD (R198A, Q200A and R207A) showed a significant reduction of RNA deaminase activity. While a co-crystal structure of APO1 with RNA and its cofactor (A1CF or RBM47) is needed to fully clarify these issues, the results described here provide important insights on how APO1 may use specific charged residues on the N-terminus as well as on the C-terminal A1HD to enable its activity on RNA substrates.

Our structure and mutational data reveal how APO1 forms a stable dimer complex mediated by the two C-terminal A1HDs, which is consistent with multiple previous biochemical and functional studies that suggested dimer formation (15,42–44). However, the functional purpose of dimerization inside cells remains unclear. One possible role may be related to regulating protein aggregation/solubility: h6 of the core structure and the A1HD are largely hydrophobic, with multiple hydrophobic residues that are packed inside buried areas as well as surface exposed to solvent, and dimerization through hydrophobic interactions effectively shields most of these surface-exposed hydrophobic residues to effectively reduce random aggregation. The C-terminal A1HD was shown here as well as by others (43) to be critical for RNA deamination. We also show that a monomeric mutant L173Q completely disrupted APO1 dimerization yet still had WT-level RNA deaminase activity. This clearly indicates that dimerization is not required for RNA editing, and a recent cellular study shown in a bioRxiv manuscript corroborates this conclusion (44).

Given the importance of the C-terminal A1HD in possibly interacting with an RNA substrate directly, it is possible that dimerization and binding to a cofactor (A1CF or RBM47) during RNA editing are mutually exclusive events. A previous model proposed for the mechanism of RNA editing by APO1 and its cofactor A1CF is that APO1 cofactor recognizes a targeted RNA through specific structural features of the RNA and then melts the structured RNA to present an unfolded RNA region to APO1 active site for deamination (45). The detailed molecular interaction between APO1, A1CF and RNA will require future structural and functional investigations, and it remains to be seen whether the large multimeric species seen *in vitro* is relevant to the high molecular mass APO1 editosomes observed inside cells (81). It is worth noting that both APO1 cofactors A1CF and RBM47 have sequence and biochemical features that bear a close resemblance to the large family of proteins commonly found within the biologically relevant phase-separated aggregates inside cells (82). Thus, it is intriguing to posit that the aggregation in the presence of APO1 cofactors seen *in vitro* and inside cells may play a role in regulating activity, storage or subcellular localization of APO1 editosomes in a similar manner as the phase-separated aggregates reported for many other systems.

In summary, we have determined the crystal structure of APO1 that reveals a canonical deaminase core structure and an extra C-terminal A1HD domain fold unique to APO1. The structure shows how APO1 dimerizes through its A1HD domain through hydrophobic interactions. The stable dimer formation can be disrupted by mutating the interface, but dimerization does not appear to be required for APO1 cofactor binding or deamination on RNA or DNA substrates. The results from the subsequent structure-guided mutational and functional studies using biochemical and cell-based assays provide new insights into the deaminase activity on both RNA and ssDNA substrates and the importance of this C-terminal domain in regulating these enzymatic functions. There are still many interesting questions with regard to how APO1 interacts with its cofactors and recognizes specific target RNA for deamination and other potential biological functions of APO1. The results reported here provide a strong structural foundation for addressing these questions in the future.

## DATA AVAILABILITY

Coordinates and structure factors have been deposited in the Protein Data Bank with PDB accession code 6X91.

## Supplementary Material

zcaa027_Supplemental_File

## References

[1] Goila-Gaur R., Strebel K. HIV-1 Vif, APOBEC, and intrinsic immunity. Retrovirology. 2008; 5:51. 18577210 10.1186/1742-4690-5-51 PMC2443170

[2] Peled J.U., Kuang F.L., Iglesias-Ussel M.D., Roa S., Kalis S.L., Goodman M.F., Scharff M.D. The biochemistry of somatic hypermutation. Annu. Rev. Immunol. 2008; 26:481–511. 18304001 10.1146/annurev.immunol.26.021607.090236

[3] Harris R.S., Petersen-Mahrt S.K., Neuberger M.S. RNA editing enzyme APOBEC1 and some of its homologs can act as DNA mutators. Mol. Cell. 2002; 10:1247–1253. 12453430 10.1016/s1097-2765(02)00742-6

[4] Petersen-Mahrt S.K., Neuberger M.S. *In vitro* deamination of cytosine to uracil in single-stranded DNA by apolipoprotein B editing complex catalytic subunit 1 (APOBEC1). J. Biol. Chem. 2003; 278:19583–19586. 12697753 10.1074/jbc.C300114200

[5] Muramatsu M., Sankaranand V.S., Anant S., Sugai M., Kinoshita K., Davidson N.O., Honjo T. Specific expression of activation-induced cytidine deaminase (AID), a novel member of the RNA-editing deaminase family in germinal center B cells. J. Biol. Chem. 1999; 274:18470–18476. 10373455 10.1074/jbc.274.26.18470

[6] Muto T., Muramatsu M., Taniwaki M., Kinoshita K., Honjo T. Isolation, tissue distribution, and chromosomal localization of the human activation-induced cytidine deaminase (AID) gene. Genomics. 2000; 68:85–88. 10950930 10.1006/geno.2000.6268

[7] Madsen P., Anant S., Rasmussen H.H., Gromov P., Vorum H., Dumanski J.P., Tommerup N., Collins J.E., Wright C.L., Dunham I. et al. Psoriasis upregulated phorbolin-1 shares structural but not functional similarity to the mRNA-editing protein apobec-1. J. Invest. Dermatol. 1999; 113:162–169. 10469298 10.1046/j.1523-1747.1999.00682.x

[8] Jarmuz A., Chester A., Bayliss J., Gisbourne J., Dunham I., Scott J., Navaratnam N. An anthropoid-specific locus of orphan C to U RNA-editing enzymes on chromosome 22. Genomics. 2002; 79:285–296. 11863358 10.1006/geno.2002.6718

[9] Sheehy A.M., Gaddis N.C., Choi J.D., Malim M.H. Isolation of a human gene that inhibits HIV-1 infection and is suppressed by the viral Vif protein. Nature. 2002; 418:27. 12167863 10.1038/nature00939

[10] Wedekind J.E., Dance G.S.C., Sowden M.P., Smith H.C. Messenger RNA editing in mammals: new members of the APOBEC family seeking roles in the family business. Trends Genet. 2003; 19:207–216. 12683974 10.1016/S0168-9525(03)00054-4

[11] Liao W., Hong S.H., Chan B.H.J., Rudolph F.B., Clark S.C., Chan L. APOBEC-2, a cardiac- and skeletal muscle-specific member of the cytidine deaminase supergene family. Biochem. Biophys. Res. Commun. 1999; 260:398–404. 10403781 10.1006/bbrc.1999.0925

[12] Anant S., Mukhopadhyay D., Sankaranand V., Kennedy S., Henderson J.O., Davidson N.O. ARCD-1, an apobec-1-related cytidine deaminase, exerts a dominant negative effect on C to U RNA editing. Am. J. Physiol. Cell Physiol. 2001; 281:C1904–C1916. 11698249 10.1152/ajpcell.2001.281.6.C1904

[13] Rogozin I.B., Basu M.K., Jordan I.K., Pavlov Y.I., Koonin E.V. APOBEC4, a new member of the AID/APOBEC family of polynucleotide (deoxy)cytidine deaminases predicted by computational analysis. Cell Cycle. 2005; 4:1281–1285. 16082223 10.4161/cc.4.9.1994

[14] Chen S.H., Habib G., Yang C.Y., Gu Z.W., Lee B.R., Weng S.A., Silberman S.R., Cai S.J., Deslypere J.P., Rosseneu M. et al. Apolipoprotein B-48 is the product of a messenger RNA with an organ-specific in-frame stop codon. Science. 1987; 238:363–366. 3659919 10.1126/science.3659919

[15] Teng B., Burant C., Davidson N. Molecular cloning of an apolipoprotein B messenger RNA editing protein. Science. 1993; 260:1816–1819. 8511591 10.1126/science.8511591

[16] Niavarani A., Shahrabi Farahani A., Sharafkhah M., Rassoulzadegan M. Pancancer analysis identifies prognostic high-APOBEC1 expression level implicated in cancer in-frame insertions and deletions. Carcinogenesis. 2018; 39:327–335. 29346513 10.1093/carcin/bgy005

[17] Yamanaka S., Balestra M.E., Ferrell L.D., Fan J., Arnold K.S., Taylor S., Taylor J.M., Innerarity T.L. Apolipoprotein B mRNA-editing protein induces hepatocellular carcinoma and dysplasia in transgenic animals. Proc. Natl Acad. Sci. U.S.A. 1995; 92:8483–8487. 7667315 10.1073/pnas.92.18.8483 PMC41181

[18] Mukhopadhyay D., Anant S., Lee R.M., Kennedy S., Viskochil D., Davidson N.O. C→U editing of neurofibromatosis 1 mRNA occurs in tumors that express both the type II transcript and apobec-1, the catalytic subunit of the apolipoprotein B mRNA-editing enzyme. Am. J. Hum. Genet. 2002; 70:38–50. 11727199 10.1086/337952 PMC384902

[19] Saraconi G., Severi F., Sala C., Mattiuz G., Conticello S.G. The RNA editing enzyme APOBEC1 induces somatic mutations and a compatible mutational signature is present in esophageal adenocarcinomas. Genome Biol. 2014; 15:417. 25085003 10.1186/s13059-014-0417-z PMC4144122

[20] Rogozin I.B., Roche-Lima A., Lada A.G., Belinky F., Sidorenko I.A., Glazko G.V., Babenko V.N., Cooper D.N., Pavlov Y.I. Nucleotide weight matrices reveal ubiquitous mutational footprints of AID/APOBEC deaminases in human cancer genomes. Cancers (Basel). 2019; 11:211. 10.3390/cancers11020211 PMC6406962 30759888

[21] Prochnow C., Bransteitter R., Klein M.G., Goodman M.F., Chen X.S. The APOBEC-2 crystal structure and functional implications for the deaminase AID. Nature. 2007; 445:447–451. 17187054 10.1038/nature05492

[22] Vasudevan A.A., Smits S.H., Hoppner A., Haussinger D., Koenig B.W., Munk C. Structural features of antiviral DNA cytidine deaminases. Biol. Chem. 2013; 394:1357–1370. 23787464 10.1515/hsz-2013-0165

[23] Navaratnam N., Morrison J.R., Bhattacharya S., Patel D., Funahashi T., Giannoni F., Teng B.B., Davidson N.O., Scott J. The p27 catalytic subunit of the apolipoprotein B mRNA editing enzyme is a cytidine deaminase. J. Biol. Chem. 1993; 268:20709–20712. 8407891

[24] Sharma S., Patnaik S.K., Taggart R.T., Baysal B.E. The double-domain cytidine deaminase APOBEC3G is a cellular site-specific RNA editing enzyme. Sci. Rep. 2016; 6:39100. 27974822 10.1038/srep39100 PMC5156925

[25] Sharma S., Patnaik S.K., Taggart R.T., Kannisto E.D., Enriquez S.M., Gollnick P., Baysal B.E. APOBEC3A cytidine deaminase induces RNA editing in monocytes and macrophages. Nat. Commun. 2015; 6:6881. 25898173 10.1038/ncomms7881 PMC4411297

[26] Meier J.C., Henneberger C., Melnick I., Racca C., Harvey R.J., Heinemann U., Schmieden V., Grantyn R. RNA editing produces glycine receptor α3P185L, resulting in high agonist potency. Nat. Neurosci. 2005; 8:736–744. 15895087 10.1038/nn1467

[27] Skuse G.R., Cappione A.J., Sowden M., Metheny L.J., Smith H.C. The neurofibromatosis type I messenger RNA undergoes base-modification RNA editing. Nucleic Acids Res. 1996; 24:478–485. 8602361 10.1093/nar/24.3.478 PMC145654

[28] Blanc V., Park E., Schaefer S., Miller M., Lin Y., Kennedy S., Billing A.M., Hamidane H.B., Graumann J., Mortazavi A. et al. Genome-wide identification and functional analysis of Apobec-1-mediated C-to-U RNA editing in mouse small intestine and liver. Genome Biol. 2014; 15:R79. 24946870 10.1186/gb-2014-15-6-r79 PMC4197816

[29] Rosenberg B.R., Hamilton C.E., Mwangi M.M., Dewell S., Papavasiliou F.N. Transcriptome-wide sequencing reveals numerous APOBEC1 mRNA-editing targets in transcript 3′ UTRs. Nat. Struct. Mol. Biol. 2011; 18:230–238. 21258325 10.1038/nsmb.1975 PMC3075553

[30] Mehta A., Kinter M.T., Sherman N.E., Driscoll D.M. Molecular cloning of apobec-1 complementation factor, a novel RNA-binding protein involved in the editing of apolipoprotein B mRNA. Mol. Cell. Biol. 2000; 20:1846–1854. 10669759 10.1128/mcb.20.5.1846-1854.2000 PMC85365

[31] Fossat N., Tourle K., Radziewic T., Barratt K., Liebhold D., Studdert J.B., Power M., Jones V., Loebel D.A.F., Tam P.P.L. C to U RNA editing mediated by APOBEC1 requires RNA-binding protein RBM47. EMBO Rep. 2014; 15:903–910. 24916387 10.15252/embr.201438450 PMC4197048

[32] Blanc V., Xie Y., Kennedy S., Riordan J.D., Rubin D.C., Madison B.B., Mills J.C., Nadeau J.H., Davidson N.O. APOBEC1 complementation factor (A1CF) and RBM47 interact in tissue-specific regulation of C to U RNA editing in mouse intestine and liver. RNA. 2019; 25:70–81. 30309881 10.1261/rna.068395.118 PMC6298562

[33] Wolfe A.D., Arnold D.B., Chen X. Comparison of RNA editing activity of APOBEC1–A1CF and APOBEC1–RBM47 complexes reconstituted in HEK293T cells. J. Mol. Biol. 2019; 431:1506–1517. 30844405 10.1016/j.jmb.2019.02.025 PMC6443457

[34] Fossat N., Radziewic T., Jones V., Tourle K., Tam P.P. Conditional restoration and inactivation of Rbm47 reveal its tissue-context requirement for viability and growth. Genesis. 2016; 54:115–122. 26789794 10.1002/dvg.22920

[35] Snyder E.M., McCarty C., Mehalow A., Svenson K.L., Murray S.A., Korstanje R., Braun R.E. APOBEC1 complementation factor (A1CF) is dispensable for C-to-U RNA editing *in vivo*. RNA. 2017; 23:457–465. 28069890 10.1261/rna.058818.116 PMC5340909

[36] Kim Y.E., Won M., Lee S.G., Park C., Song C.H., Kim K.K. RBM47-regulated alternative splicing of TJP1 promotes actin stress fiber assembly during epithelial-to-mesenchymal transition. Oncogene. 2019; 38:6521–6536. 31358901 10.1038/s41388-019-0892-5

[37] Radine C., Peters D., Reese A., Neuwahl J., Budach W., Janicke R.U., Sohn D. The RNA-binding protein RBM47 is a novel regulator of cell fate decisions by transcriptionally controlling the p53–p21-axis. Cell Death Differ. 2020; 27:1274–1285. 31511650 10.1038/s41418-019-0414-6 PMC7206044

[38] Rokavec M., Kaller M., Horst D., Hermeking H. Pan-cancer EMT-signature identifies RBM47 down-regulation during colorectal cancer progression. Sci. Rep. 2017; 7:4687. 28680090 10.1038/s41598-017-04234-2 PMC5498532

[39] Ito F., Fu Y., Kao S.C.A., Yang H., Chen X.S. Family-wide comparative analysis of cytidine and methylcytidine deamination by eleven human APOBEC proteins. J. Mol. Biol. 2017; 429:1787–1799. 28479091 10.1016/j.jmb.2017.04.021 PMC5530319

[40] Krishnan A., Iyer L.M., Holland S.J., Boehm T., Aravind L. Diversification of AID/APOBEC-like deaminases in metazoa: multiplicity of clades and widespread roles in immunity. Proc. Natl Acad. Sci. U.S.A. 2018; 4:201720897. 10.1073/pnas.1720897115 PMC5889660 29555751

[41] Severi F., Chicca A., Conticello S.G. Analysis of reptilian APOBEC1 suggests that RNA editing may not be its ancestral function. Mol. Biol. Evol. 2011; 28:1125–1129. 21172829 10.1093/molbev/msq338

[42] Lau P.P., Zhu H.J., Baldini A., Charnsangavej C., Chan L. Dimeric structure of a human apolipoprotein B mRNA editing protein and cloning and chromosomal localization of its gene. Proc. Natl Acad. Sci. U.S.A. 1994; 91:8522–8526. 8078915 10.1073/pnas.91.18.8522 PMC44638

[43] Teng B.B., Ochsner S., Zhang Q., Soman K.V., Lau P.P., Chan L. Mutational analysis of apolipoprotein B mRNA editing enzyme (APOBEC1): structure–function relationships of RNA editing and dimerization. J. Lipid Res. 1999; 40:623–635. 10191286

[44] Chieca M., Montini M., Severi F., Pecori R., Conticello S.G. Dimerisation of APOBEC1 is dispensable for its RNA editing activity. 2018; bioRxiv doi:06 September 2018,preprint: not peer reviewed 10.1101/410803.

[45] Maris C., Masse J., Chester A., Navaratnam N., Allain F.H.-T. NMR structure of the apoB mRNA stem-loop and its interaction with the C to U editing APOBEC1 complementary factor. RNA. 2005; 11:173–186. 15659357 10.1261/rna.7190705 PMC1370706

[46] Grunewald J., Zhou R., Garcia S.P., Iyer S., Lareau C.A., Aryee M.J., Joung J.K. Transcriptome-wide off-target RNA editing induced by CRISPR-guided DNA base editors. Nature. 2019; 569:433–437. 30995674 10.1038/s41586-019-1161-z PMC6657343

[47] Bienert S., Waterhouse A., De Beer T.A.P., Tauriello G., Studer G., Bordoli L., Schwede T. The SWISS-MODEL Repository: new features and functionality. Nucleic Acids Res. 2017; 45:D313–D319. 27899672 10.1093/nar/gkw1132 PMC5210589

[48] Guex N., Peitsch M.C., Schwede T. Automated comparative protein structure modeling with SWISS-MODEL and Swiss-PdbViewer: a historical perspective. Electrophoresis. 2009; 30:S162–S173. 19517507 10.1002/elps.200900140

[49] Waterhouse A., Bertoni M., Bienert S., Studer G., Tauriello G., Gumienny R., Heer F.T., De Beer T.A.P., Rempfer C., Bordoli L. et al. SWISS-MODEL: homology modelling of protein structures and complexes. Nucleic Acids Res. 2018; 46:W296–W303. 29788355 10.1093/nar/gky427 PMC6030848

[50] Ito F., Yang H., Xiao X., Li S.X., Wolfe A., Zirkle B., Arutiunian V., Chen X.S. Understanding the structure, multimerization, subcellular localization and mC selectivity of a genomic mutator and anti-HIV factor APOBEC3H. Sci. Rep. 2018; 8:3763. 29491387 10.1038/s41598-018-21955-0 PMC5830531

[51] Porter J.R., Weitzner B.D., Lange O.F. A framework to simplify combined sampling strategies in Rosetta. PLoS One. 2015; 10:e0138220. 26381271 10.1371/journal.pone.0138220 PMC4575156

[52] Fleishman S.J., Leaver-Fay A., Corn J.E., Strauch E.M., Khare S.D., Koga N., Ashworth J., Murphy P., Richter F., Lemmon G. et al. RosettaScripts: a scripting language interface to the Rosetta macromolecular modeling suite. PLoS One. 2011; 6:e20161. 21731610 10.1371/journal.pone.0020161 PMC3123292

[53] Leaver-Fay A., Tyka M., Lewis S.M., Lange O.F., Thompson J., Jacak R., Kaufman K., Renfrew P.D., Smith C.A., Sheffler W. et al. Rosetta3: an object-oriented software suite for the simulation and design of macromolecules. Methods Enzymol. 2011; 487:545–574. 21187238 10.1016/B978-0-12-381270-4.00019-6 PMC4083816

[54] Kim D.E., Chivian D., Baker D. Protein structure prediction and analysis using the Robetta server. Nucleic Acids Res. 2004; 32:W526–W531. 15215442 10.1093/nar/gkh468 PMC441606

[55] Lehmann D.M., Galloway C.A., MacElrevey C., Sowden M.P., Wedekind J.E., Smith H.C. Functional characterization of APOBEC-1 complementation factor phosphorylation sites. Biochim. Biophys. Acta Mol. Cell Res. 2007; 1773:408–418. 10.1016/j.bbamcr.2006.11.019 PMC1847399 17229474

[56] Otwinowski Z., Minor W. Processing of X-ray diffraction data collected in oscillation mode. Methods Enzymol. 1997; 276:306–315. 10.1016/S0076-6879(97)76066-X 27754618

[57] Adams P.D., Afonine P.V., Bunkóczi G., Chen V.B., Davis I.W., Echols N., Headd J.J., Hung L.W., Kapral G.J., Grosse-Kunstleve R.W. et al. PHENIX: a comprehensive Python-based system for macromolecular structure solution. Acta Crystallogr. D. 2010; 66:213–221. 20124702 10.1107/S0907444909052925 PMC2815670

[58] Quiocho F.A., Spurlino J.C., Rodseth L.E. Extensive features of tight oligosaccharide binding revealed in high-resolution structures of the maltodextrin transport/chemosensory receptor. Structure. 1997; 5:997–1015. 9309217 10.1016/s0969-2126(97)00253-0

[59] Emsley P., Lohkamp B., Scott W.G., Cowtan K. Features and development of Coot. Acta Crystallogr. D. 2010; 66:486–501. 20383002 10.1107/S0907444910007493 PMC2852313

[60] Driscoll D., Wynne J., Wallis S., Scott J. An *in vitro* system for the editing of apolipoprotein B mRNA. Cell. 1989; 58:519–525. 2758465 10.1016/0092-8674(89)90432-7

[61] Ryder S.P., Recht M.I., Williamson J.R. Quantitative analysis of protein–RNA interactions by gel mobility shift. Methods Mol. Biol. 2008; 488:99–115. 18982286 10.1007/978-1-60327-475-3_7 PMC2928675

[62] Schindelin J., Arganda-Carreras I., Frise E., Kaynig V., Longair M., Pietzsch T., Preibisch S., Rueden C., Saalfeld S., Schmid B. et al. Fiji: an open-source platform for biological-image analysis. Nat. Methods. 2012; 9:676–682. 22743772 10.1038/nmeth.2019 PMC3855844

[63] Qiao Q., Wang L., Meng F.L., Hwang J.K., Alt F.W., Wu H. AID recognizes structured DNA for class switch recombination. Mol. Cell. 2017; 67:361–373. 28757211 10.1016/j.molcel.2017.06.034 PMC5771415

[64] Jacobs W.M., Shakhnovich E.I. Structure-based prediction of protein-folding transition paths. Biophys. J. 2016; 111:925–936. 27602721 10.1016/j.bpj.2016.06.031 PMC5018131

[65] Xue L.C., Rodrigues J.P., Kastritis P.L., Bonvin A.M., Vangone A. PRODIGY: a web server for predicting the binding affinity of protein–protein complexes. Bioinformatics. 2016; 32:3676–3678. 27503228 10.1093/bioinformatics/btw514

[66] Dawson N.L., Lewis T.E., Das S., Lees J.G., Lee D., Ashford P., Orengo C.A., Sillitoe I. CATH: an expanded resource to predict protein function through structure and sequence. Nucleic Acids Res. 2017; 45:D289–D295. 27899584 10.1093/nar/gkw1098 PMC5210570

[67] Navaratnam N., Fujino T., Bayliss J., Jarmuz A., How A., Richardson N., Somasekaram A., Bhattacharya S., Carter C., Scott J. *Escherichia coli* cytidine deaminase provides a molecular model for ApoB RNA editing and a mechanism for RNA substrate recognition. J. Mol. Biol. 1998; 275:695–714. 9466941 10.1006/jmbi.1997.1506

[68] Xie K., Sowden M.P., Dance G.S., Torelli A.T., Smith H.C., Wedekind J.E. The structure of a yeast RNA-editing deaminase provides insight into the fold and function of activation-induced deaminase and APOBEC-1. Proc. Natl Acad. Sci. U.S.A. 2004; 101:8114–8119. 15148397 10.1073/pnas.0400493101 PMC419566

[69] Chester A., Somasekaram A., Tzimina M., Jarmuz A., Gisbourne J., O’Keefe R., Scott J., Navaratnam N. The apolipoprotein B mRNA editing complex performs a multifunctional cycle and suppresses nonsense-mediated decay. EMBO J. 2003; 22:3971–3982. 12881431 10.1093/emboj/cdg369 PMC169042

[70] Ikeda T., Abd El Galil K.H., Tokunaga K., Maeda K., Sata T., Sakaguchi N., Heidmann T., Koito A. Intrinsic restriction activity by apolipoprotein B mRNA editing enzyme APOBEC1 against the mobility of autonomous retrotransposons. Nucleic Acids Res. 2011; 39:5538–5554. 21398638 10.1093/nar/gkr124 PMC3141244

[71] Bohn J.A., Thummar K., York A., Raymond A., Brown W.C., Bieniasz P.D., Hatziioannou T., Smith J.L. APOBEC3H structure reveals an unusual mechanism of interaction with duplex RNA. Nat. Commun. 2017; 8:2–10. 29044109 10.1038/s41467-017-01309-6 PMC5647330

[72] Matsuoka T., Nagae T., Ode H., Awazu H., Kurosawa T., Hamano A., Matsuoka K., Hachiya A., Imahashi M., Yokomaku Y. et al. Structural basis of chimpanzee APOBEC3H dimerization stabilized by double-stranded RNA. Nucleic Acids Res. 2018; 46:10368–10379. 30060196 10.1093/nar/gky676 PMC6212771

[73] Rathore A., Carpenter M.A., Demir Ö., Ikeda T., Li M., Shaban N.M., Law E.K., Anokhin D., Brown W.L., Amaro R.E. et al. The local dinucleotide preference of APOBEC3G can be altered from 5′-CC to 5′-TC by a single amino acid substitution. J. Mol. Biol. 2013; 425:4442–4454. 23938202 10.1016/j.jmb.2013.07.040 PMC3812309

[74] Shaban N.M., Shi K., Lauer K.V., Carpenter M.A., Richards C.M., Salamango D., Wang J., Lopresti M.W., Banerjee S., Levin-Klein R. et al. The antiviral and cancer genomic DNA deaminase APOBEC3H is regulated by an RNA-mediated dimerization mechanism. Mol. Cell. 2018; 69:75–86. 29290613 10.1016/j.molcel.2017.12.010 PMC5991973

[75] Shi K., Carpenter M.A., Banerjee S., Shaban N.M., Kurahashi K., Salamango D.J., McCann J.L., Starrett G.J., Duffy J.V., Demir Ö. et al. Structural basis for targeted DNA cytosine deamination and mutagenesis by APOBEC3A and APOBEC3B. Nat. Struct. Mol. Biol. 2017; 24:131–139. 27991903 10.1038/nsmb.3344 PMC5296220

[76] Kouno T., Silvas T.V., Hilbert B.J., Shandilya S.M.D., Bohn M.F., Kelch B.A., Royer W.E., Somasundaran M., Yilmaz N.K., Matsuo H. et al. Crystal structure of APOBEC3A bound to single-stranded DNA reveals structural basis for cytidine deamination and specificity. Nat. Commun. 2017; 8:15024. 28452355 10.1038/ncomms15024 PMC5414352

[77] Maiti A., Myint W., Kanai T., Delviks-Frankenberry K., Sierra Rodriguez C., Pathak V.K., Schiffer C.A., Matsuo H. Crystal structure of the catalytic domain of HIV-1 restriction factor APOBEC3G in complex with ssDNA. Nat. Commun. 2018; 9:2460. 29941968 10.1038/s41467-018-04872-8 PMC6018426

[78] Shah R.R., Knott T.J., Legros J.E., Navaratnam N., Greeve J.C., Scott J. Sequence requirements for the editing of apolipoprotein B mRNA. J. Biol. Chem. 1991; 266:16301–16304. 1885564

[79] Liu Z., Chen O., Wall J.B.J., Zheng M., Zhou Y., Wang L., Ruth Vaseghi H., Qian L., Liu J. Systematic comparison of 2A peptides for cloning multi-genes in a polycistronic vector. Sci. Rep. 2017; 7:2193. 28526819 10.1038/s41598-017-02460-2 PMC5438344

[80] Ryan M.D., King A.M.Q., Thomas G.P. Cleavage of foot-and-mouth disease virus polyprotein is mediated by residues located within a 19 amino acid sequence. J. Gen. Virol. 1991; 72:2727–2732. 1658199 10.1099/0022-1317-72-11-2727

[81] Smith H.C., Kuo S.R., Backus J.W., Harris S.G., Sparks C.E., Sparks J.D. *In vitro* apolipoprotein B mRNA editing: identification of a 27S editing complex. Proc. Natl Acad. Sci. U.S.A. 1991; 88:1489–1493. 1996349 10.1073/pnas.88.4.1489 PMC51044

[82] Banani S.F., Lee H.O., Hyman A.A., Rosen M.K. Biomolecular condensates: organizers of cellular biochemistry. Nat. Rev. Mol. Cell Biol. 2017; 18:285–298. 28225081 10.1038/nrm.2017.7 PMC7434221

