# Supplementary material for: The structure of APOBEC1 and insights into its RNA and DNA substrate selectivity
Source: NAR Cancer. 2020 Oct 9;2(4):zcaa027. doi: 10.1093/narcan/zcaa027 (PMC7556403; doi:10.1093/narcan/zcaa027)
Supplement: zcaa027_Supplemental_File [file zcaa027_supplemental_file.pdf]

Supplementary Figures and Modeling Scripts

The structure of APOBEC1 and insights into its RNA and DNA substrate selectivity

Wolfe et al.

a

Mutants	Residue mutated	Solubility
mA	F205A	Slight solubility
mB	M46A, R48S, I80T	Slight solubility
mBC	M46A, R48S, I80T, L173A	Slight solubility
mBD	M46A, R48S, I80T, W199A	Slight solubility
(mABCD) mSOL	M46A, R48S, I80T, L173A, W199A, F205A	Medium solubility
mXT (crystallized)	NΔ14, mSOL+W121A	High solubility

c

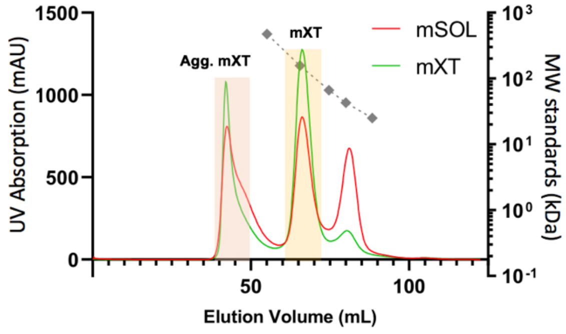

b

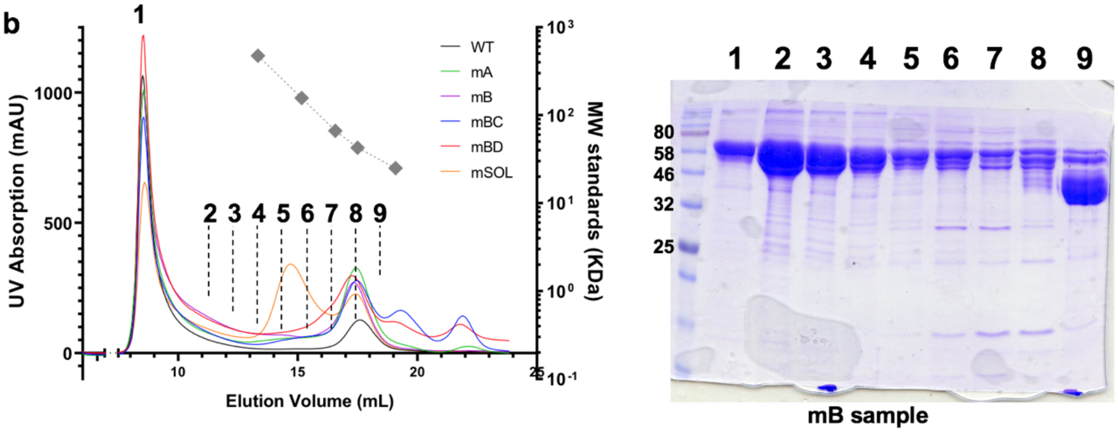

Supplementary Figure 1: Additional data from engineering a stable homogeneous APO1 construct. (a) Point mutations tested in series to generate a soluble APO1 construct for crystallization. Different mutations were made sequentially in separate rounds of purification to obtain the well-behaved mXT construct. Further mutation of the catalytic residue E63A on mXT generated the mXTi construct that yielded crystals. **(b)** An overlay of each of the six SEC profile traces shows a subtle increase in peak height relative to the WT trace around the 15 mL approximate dimeric elution volume. At right, SDS-page of fractions from the mB trace as an example shows that the MBP-APO1 eluted in all fractions down to the cleaved MBP peak in fraction 9. Combining all mutations that showed improvement in solubility into one construct, mSOL, (orange line) yielded a peak at roughly a dimeric elution volume. **(c)** Effect of the W121A mutation on improving the overall yield of MBP-APO1 mSOL. When purifying mXT, protein from the aggregated or dimeric peak was collected and concentrated separately for testing nucleic acid binding and deaminase activity.

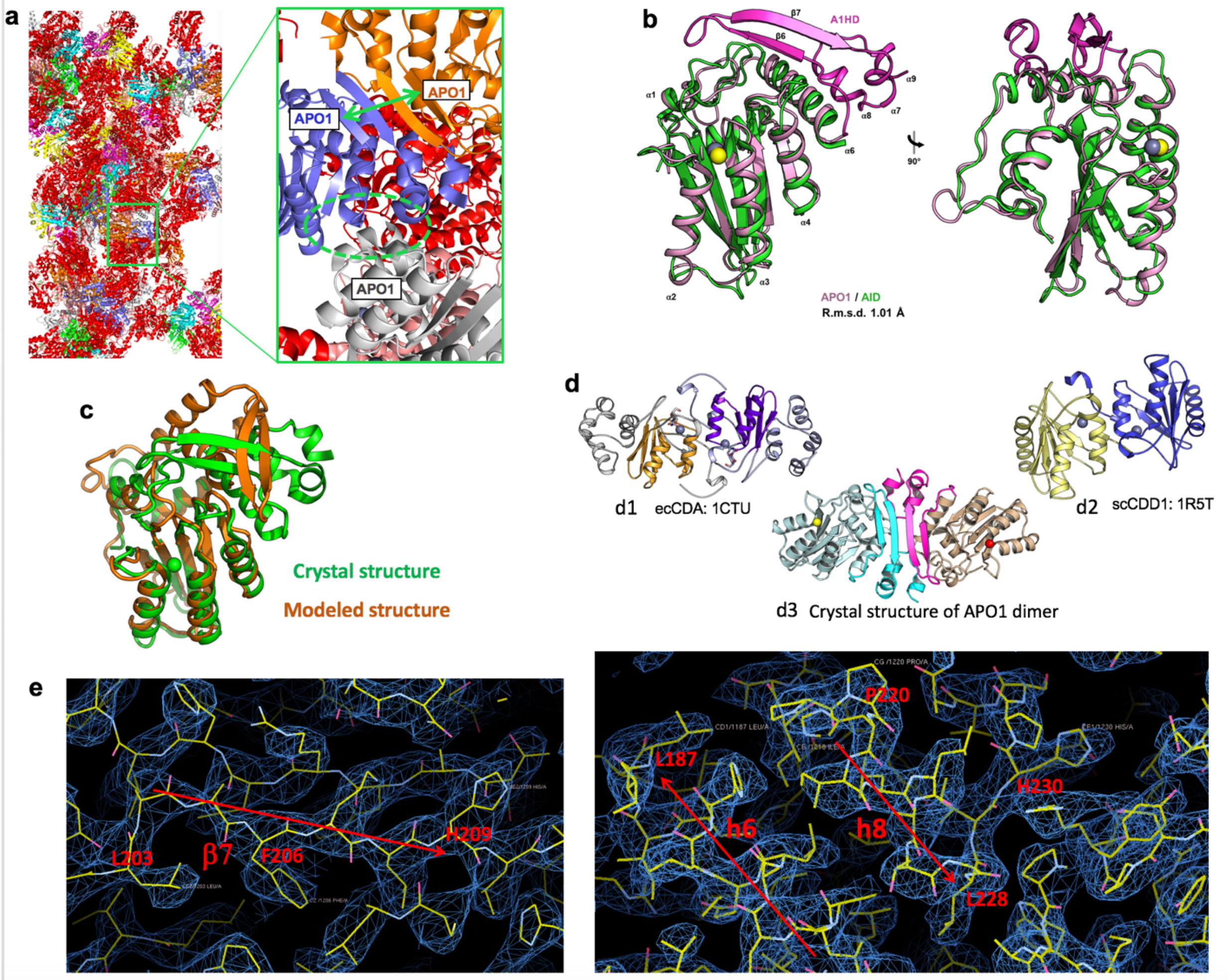

Supplementary Figure 2: Additional information about the MBP-APO1 crystal structure. (a) Left-panel: the overall view of the crystal packing of many MBP-APO1 molecules in the crystal, with all MBP drawn in red, and the APO1 moiety of the eight subunits in each asymmetric unit (asu) drawn in blue, orange, grey, and other non-red colors. The view shows that the crystal packing of the MBP-APO1 fusion is made mostly through the red-colored MBP. Right-panel: a close-up view of the boxed (in green) area from the left-panel, which shows the only two contacts existing in the crystal structure between the three APO1 molecules colored in orange, blue, and grey. The contact between the APO1 in orange and blue (or dimer interface indicated by an arrow) are extensive and has a buried surface area of about 1526.5 Å^2, which was shown to be important for stable dimer formation by mutational studies (see **Figure 5a,b** and **Supplementary Figure 5b**). However, the contact between the APO1 in blue and grey (indicated by dotted circle) has only a buried area of about 451.2 Å^2, which is too small to be considered as a relevant inter-molecular interaction (see also subpanel c). **(b)** An alignment of the core deaminase domain of APO1 (aa 15-186) (pink) with the structure of AID (PDB ID: 5W0R, in green), showing that the N-terminal core deaminase domain of APO1 aligns well with the structure of AID, with an r.m.s.d. of 1.01 Å. The C-terminal A1HD unique to APO1 is shown in hot pink. **(c)** The overlap of the crystal (green) and the modeled (salmon) structures, showing there is a close similarity of core deaminase domain part at the bottom, a significant difference in the C-terminal A1HD domain at the top. **(d)** A comparison of previously modeled APO1 dimerization based on the structures of CDA proteins from *E. coli* (d1) and *S. cerevisiae* (d2) with the APO1 crystal dimer structure (d3). **(e)** Two sections of electron density map (composite omit map) from the A1HD domain that was labeled on the left with residues L203-H209 of β7, and on the right residues P220-L228 of h8 (see panel-b for A1HD labeling), showing good density for the main and side chains. The map was drawn at 1.49 sigma level with a B sharpen factor of -100.

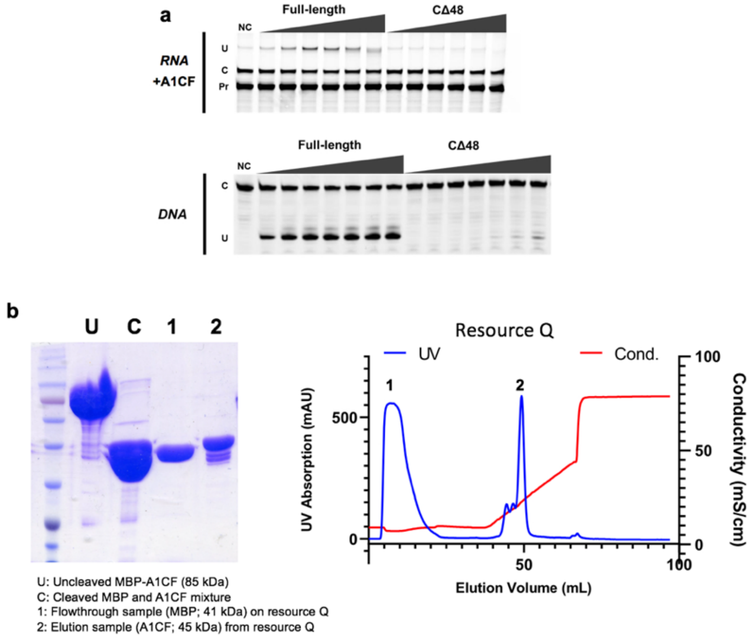

Supplementary Figure 3: Additional deamination data and A1CF purification. (a) Deletion of the last 48 residues from the C-terminus of APO1 showed diminished deamination activity both on *APOB* RNA (top) and DNA (bottom) despite of the presence of the core deaminase domain of APO1. It is possible that the removal of this C-terminal fragment may destabilize the overall cytidine deamination domain of APO1. Alternatively, this C-terminal fragment may be essential for orienting both ssDNA and RNA substrates into the correct position for deamination. **(b)** Purification of A1CF containing residues 1-391. This A1CF construct was expressed as a cleavable MBP-fusion. The SDS-PAGE on the left: lane U shows that the concentrated affinity purified uncleaved MBP-A1CF protein was very pure, lane C shows that the fusion was cleaved to completion with HRV protease to generate MBP and A1CF that migrated closely, lane 1 shows that MBP was in the flow-through peak (peak 1) of resource Q column chromatography on the right, and lane 2 shows that A1CF was in peak 2.

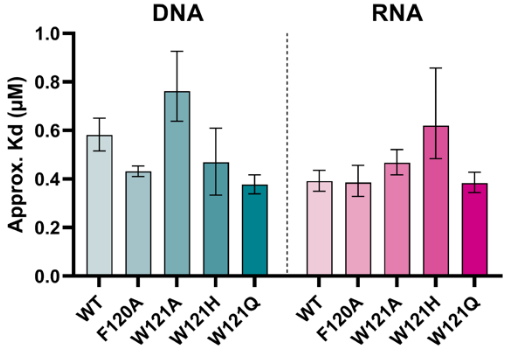

Supplementary Figure 4: Additional data for DNA and RNA binding by different APO1 mutants. Kd values for APO1 binding to the 50 nt ssDNA or 55 nt *APOB* RNA deamination substrates were estimated based on EMSA assay. Only the aggregated form of APO1 WT and mutants were used for this assay. The 50 nt ssDNA or 55 nt *APOB* RNA that served as the deamination substrates of APO1 were used. Error bars represent standard deviation calculated from three sets of experiments. The four mutants showed some variation of binding affinity to ssDNA and APOB RNA, but quite comparable to that of WT. Corresponding raw EMSA gels are provided in **Supplementary Figure 7**.

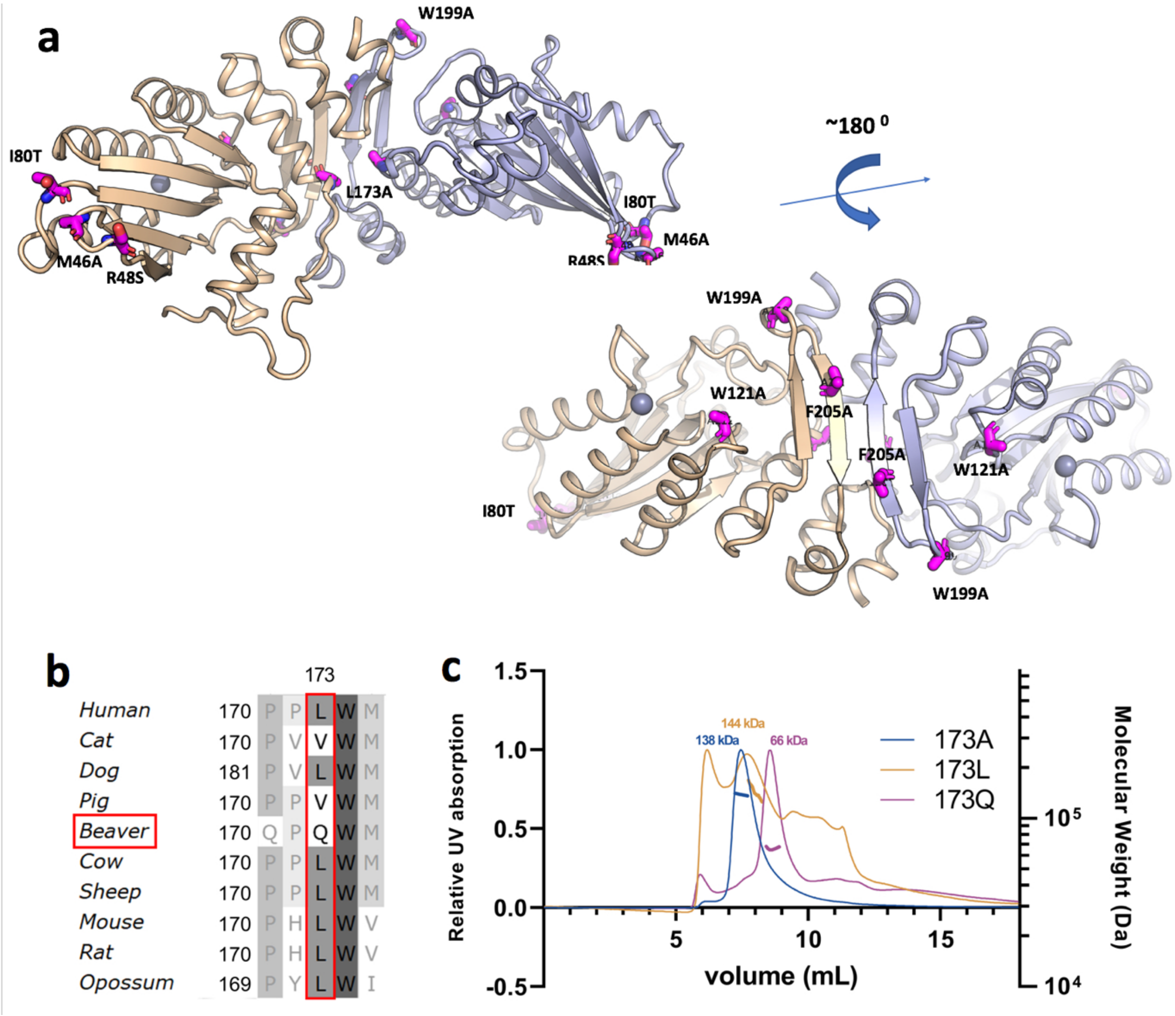

Supplementary Figure 5: Additional data for characterizing APO1 dimerization. (a) Two views of the locations of the eight mutated residues (as shown in Fig. 1b) on the APO1 dimer structure. The two views are related to each other with approximately 180° rotation. **(b)** Sequence alignment of APO1 from different organisms showing residue 173 is highly conserved and often either a leucine or valine. However, residue 173 is the larger polar residue glutamine in beaver. **(c)** Measuring the MWt of proteins of three different APO1 constructs with each containing either an alanine (173A or L173A, the original mXT construct), a leucine (173L or L173L), or glutamine (173Q or L173Q) at residue 173 by multi-angle light scattering (MALS). MALS of APO1 construct with 173A (mXT construct, blue line) showed a MWt of a dimeric APO1 of ~138kDa. Mutating residue 173 back to WT leucine (173L, yellow line) showed a broad dimer peak, with a sharp upward slope of the observed MWt, which implies that the sample tends towards larger aggregation. The trailing peaks showed variable MWt averaging about 80 kDa, further indicating the heterogeneous nature of this construct. The construct 173Q (purple line) clearly displays a smaller MWt peak measured to be ~66 kDa by MALS, which is the monomeric size of MBP-APO1.

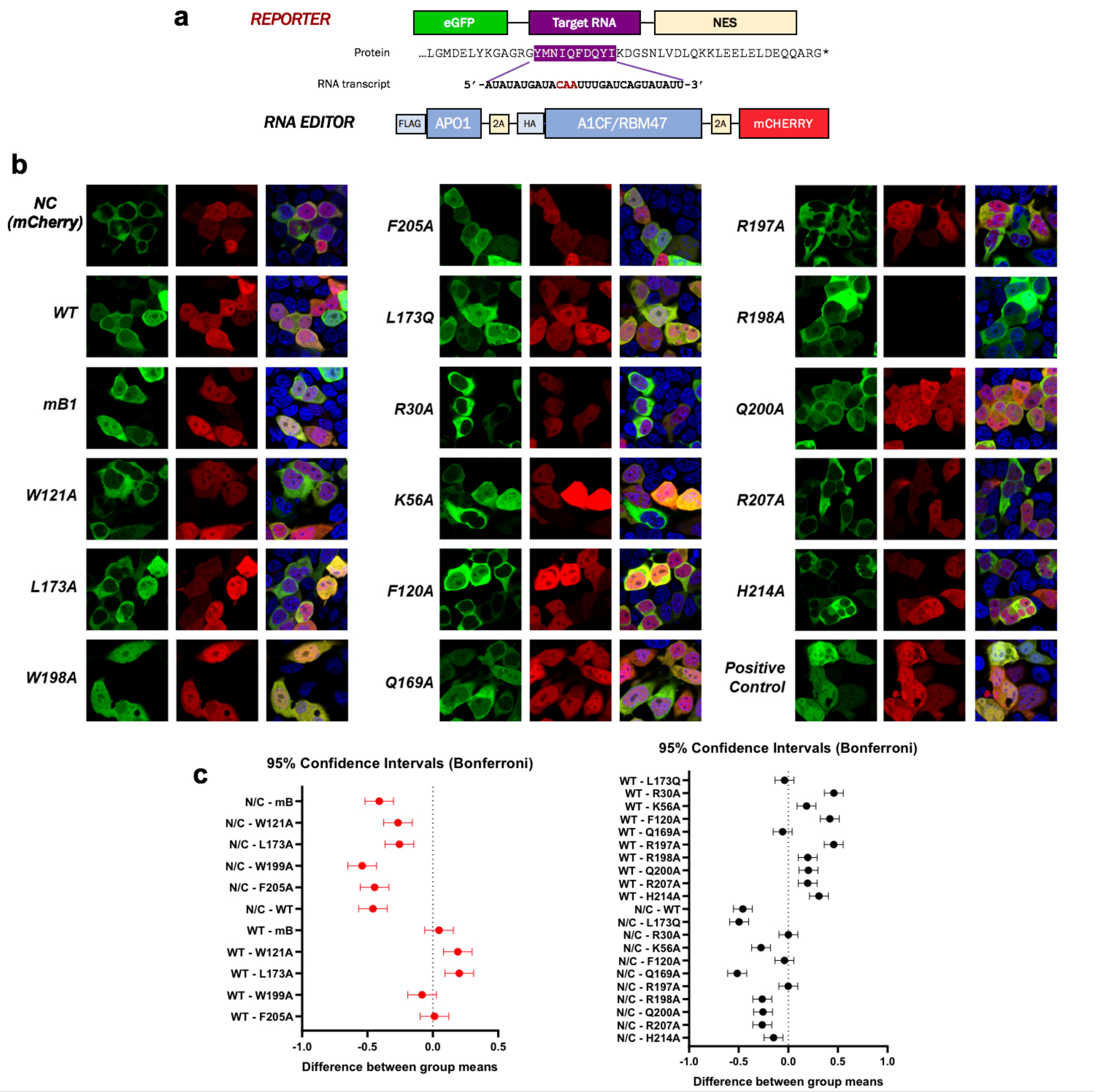

Supplementary Figure 6: Additional data supporting the cell-based fluorescence assay results shown in Figure 6. (a) Design of the reporter and editor constructs for the cell-based fluorescent assay of RNA editing activity (see Wolfe et al 2019, ref. #28). **(b)** Representative live-cell confocal fluorescence images of all samples tested. eGFP (green) is the reporter channel, and was used for quantification by assessing the relative intensity for fluorescence in the nucleus compared to the cytosol. Nuclear fluorescence should only appear when RNA editing occurs, as shown in the positive control, which has the targeted cytosine manually mutated to a thymine on the DNA, creating a stop codon as in the case of RNA editing. 25–50 cells per sample were imaged and quantified. mCherry (red) is used as a reporter for the expression of the upstream APO1 and A1CF (28). Hoechst 33342 (blue) was used to stain genomic DNA to define the nuclei and can be seen in the composite image of all three channels for each experiment. Note that in the R198A sample, mCherry was later found to have an inactivating mutation resulting in no such fluorescence. **(c)** Plot of the calculated confidence intervals from the ANOVA assessments described in Figure 6. These plots show the likelihood that the means of two different tests are significantly different, and are included to provide the rest of the comparisons not shown in Figure 6 (for which only the comparison with wildtype is shown). If the horizontal bars indicating the 95% confidence interval for a comparison intersects 0.0, those two means are not statistically significant ($P < 0.05$). At left is the comparison from Figure 6b; at right is Figure 6d.

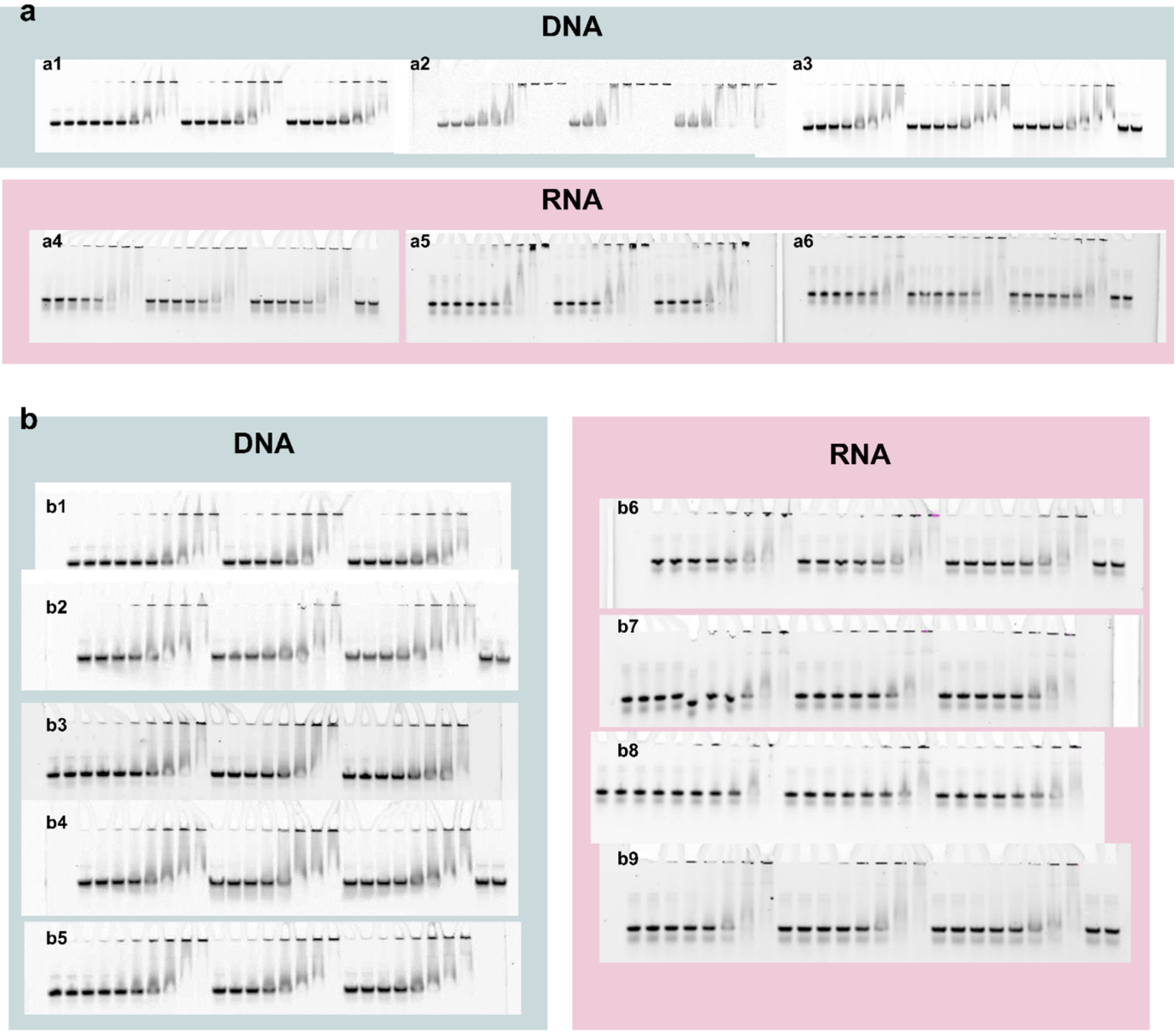

Supplementary Figure 7: Additional data for DNA and RNA binding by different APO1 mutants. (a) Raw EMSA gels for binding to FAM-50 DNA by (a1) WT, (a2) mXT, and (a3) mXT-aggregated and APOB RNA binding to (a4) WT, (a5) mXT, and (a6) mXT-aggregated. Kd values for APO1 binding to the 50 nt ssDNA or 55 nt *APOB* RNA deamination substrates (shown in Figure 3e) were estimated based on EMSA assay by fitting the % substrate unbound. **(b)** The corresponding raw EMSA gels for Supplementary Figure 4 are shown, with DNA binding by (b2) WT (a repeat of before), (b3) F120A, (b4) W121A, (b5) W121H, and (b6) W121Q and RNA binding by (b7) F120A, (b8) W121A, (b9) W121H and (b10) W121Q. Only the aggregated form of APO1 WT and mutants were used for this assay.

Supplementary File 1: Rosetta input flags and XML script.

```
################################################################################
######              This is the input flags file.                    ######
################################################################################

## Rosetta is run within the input directory with the command:
$ [rosetta-scripts] @flags -nstruct 1000

## main flags
-parser:protocol fixed_core.xml                    # input xml (see below)
#-parser:view                                      # for visualization
#-in:file:fasta input/apo1-input2.fasta            # not used
-in:file:s input/apo1-try5_04.pdb                    # used pdb input
-out:file:silent output/fixed_core.out
-out:file:silent_struct_type protein

## used these for debugging the fragments
-out:levels protocols.environment:debug
-out:levels protocols.environment.EnvClaimBroker:debug
-out:levels protocols.environment.EnvMover:debug
-out:levels protocols.environment.movers.FragmentCM:debug
-out:levels core.environment:debug
-out:levels core.pose:debug
-out:levels core.fragment:debug
-out:levels protocols.jd2:debug
-out:levels protocols.abinitio.abscript.RigidChunkCM:debug
-out:levels core.kinematics.MoveMap:debug

## denovo_flags
## most of this came from the Topo Broker tutorial
-abrelax true
-jumps:ramp_chainbreaks
-jumps:overlap_chainbreak
# magic energy fixes
-rsd_wt_helix 0.5
-rsd_wt_loop 0.5
-rg_reweight 0.5
# for loop closing
-overwrite_filter_scorefxn score3
-detect_disulf false
# loop-closing filter in SlidingWindow
-fast_loops:overwrite_filter_scorefxn score3

################################################################################
######  This is the input XML file. It provides the protocol for running ######
######          rosetta-scripts and calling the Topology Broker.        ######
################################################################################

<ROSETTASCRIPTS>
  <RESIDUE_SELECTORS>
    <Index name="apo1-core" resnums="1-157" />
    <Index name="apo1-cterm" resnums="158-222" />
  </RESIDUE_SELECTORS>
  <MOVERS>
    <SwitchResidueTypeSetMover name="centroid" set="centroid" />
    <RigidChunkCM name="chunk" selector="apo1-core"
```

```
                region_selector="apo1-core" template="INPUT"/>
    <AbscriptLoopCloserCM name="closer" fragments="input/apo1-cterm.frag3" />
    <AbscriptMover name="abinitio" cycles=5 >
      <Fragments selector="apo1-cterm" small_frags="input/apo1-cterm.frag3"
          large_frags="input/apo1-cterm.frag9" />
      <Stage ids="I-IVb" >
      </Stage>
    </AbscriptMover>
    <Environment name="env" auto_cut=1 allow_pure_movers=1>
      <Register mover="chunk" />
      <Apply mover="abinitio" />
      <Apply mover="closer" />
    </Environment>
    <Idealize name="idealize"/>
    <SwitchResidueTypeSetMover name="fullatom" set="fa_standard" />
    <FastRelax name="relax" repeats=5 />
  </MOVERS>
  <FILTERS>
  </FILTERS>
  <PROTOCOLS>
    <Add mover="centroid" />
    <Add mover="env" />
    <Add mover="idealize" />
    <Add mover="fullatom" />
    <Add mover="relax" />
  </PROTOCOLS>
</ROSETTASCRIPTS>
```
